# Standing genetic variation facilitates climatic adaptation in the invasive grapevine pathogen *Plasmopara viticola* under demographic bottlenecks

**DOI:** 10.1016/j.isci.2026.117847

**Published:** 2026-10-14

**Authors:** Xuncheng Wang, Yonghua Li, Junbo Peng, Wei Zhang, Ishara S. Manawasinghe, Qikai Xing, Hui Wang, Mei Liu, Ying Zhou, Linna Wu, Jianping Xu, Xinghong Li, Jiye Yan

**Affiliations:** 1 Key Laboratory of Environment Friendly Management on Fruit and Vegetable Pests in North China (Co-construction by Ministry and Province), Ministry of Agriculture and Rural Affairs, Institute of Plant Protection, Beijing Academy of Agriculture and Forestry Sciences, Beijing 100097, China; 2 Department of Biology, Faculty of Science, McMaster University, Hamilton, ON L8S 4K1, Canada

**Keywords:** biogeography, population genomics, gene flow, genetic load, purifying selection, founder effect

## Abstract

Invasive pathogens often experience demographic bottlenecks during colonization, yet how they rapidly adapt remains unclear. *Plasmopara viticola*, the causal agent of grapevine downy mildew, provides a model to address this question. Using β-tubulin sequences and whole-genome data, we reconstructed its invasion history and adaptation in China. Our analyses support a North American ancestral origin of Chinese *P. viticola*, while demographic modeling and ABBA-BABA tests indicate historical connections between Chinese and European-related lineages, suggesting a complex invasion history. Chinese populations showed reduced genetic diversity and regional bottlenecks, with the low-latitude population showing the most pronounced reduction in effective population size. Despite these constraints, candidate adaptive variants were frequently retained as polymorphisms in other populations and occurred at relatively high frequencies, supporting an important role for selection on pre-existing genetic variation during climatic adaptation. These findings highlight how invasive pathogens can retain adaptive potential despite severe founder effects.

## Highlights

• Chinese *P. viticola* shows North American ancestry and a complex invasion history
• Demographic analyses reveal historical links with European-related lineages in China
• Chinese populations experienced heterogeneous introduction and founder histories
• Standing genetic variation facilitated climatic adaptation despite bottlenecks

## Graphical abstract

**Figure:**
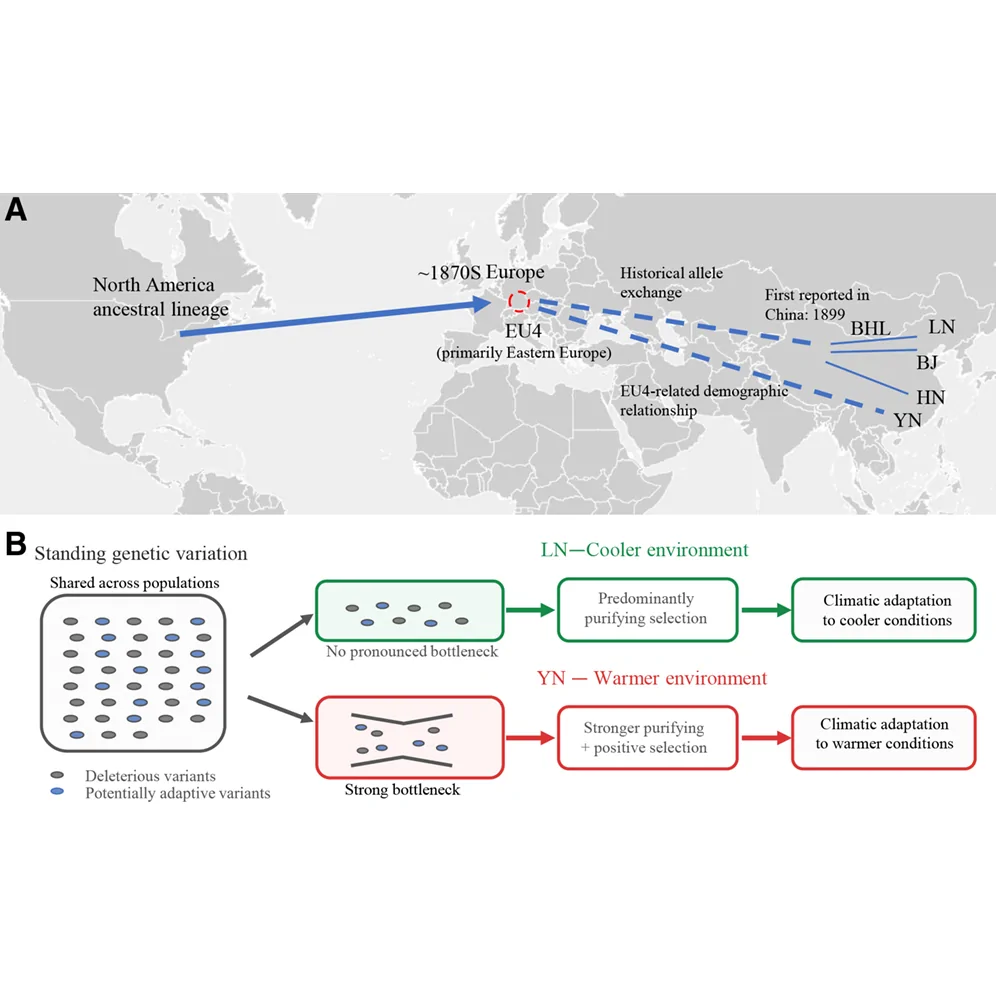

## Teaser

Microbiology; Plant population biology; Plant Genetics

## Introduction

Plant pathogens represent a critical challenge to global food security and ecosystem stability by causing widespread disease outbreaks that compromise agricultural productivity and threaten plant biodiversity across diverse ecosystems.^1^ The ongoing expansion of global plant trade and climate change has facilitated the spread and adaptation of plant pathogens into new ecological niches and geographic regions, resulting in severe disease epidemics worldwide.^2^ To mitigate the impact of emerging pathogens, it is crucial to understand their origins and the genetic mechanisms that facilitate their successful establishment outside their native range.^3^

When a pathogen is introduced into a new region, the invading population is often small.^4^ A small founder population may fail to transfer all source alleles to the new population, resulting in reduced genetic diversity compared with the source population.^5^^,^^6^ Decreased genetic diversity can lead to inbreeding, which increases homozygosity of partially recessive deleterious alleles and reduces heterozygosity at loci with heterozygote advantage, ultimately leading to fitness loss or inbreeding depression.^7^^,^^8^ This presents a paradox for invasive species that successfully colonize new territories despite the reduced genetic diversity of their small founding populations.^9^ Recent genetic studies suggest that invasive populations may maintain genetic diversity through large founding populations, repeated introductions, or gene flow, thereby addressing the genetic paradox underlying biological invasions.^10^^,^^11^^,^^12^^,^^13^ In populations with reduced genetic diversity, the expression of deleterious recessive alleles may increase. However, natural selection can act to eliminate these alleles, potentially enhancing resilience to bottlenecks and inbreeding depression during the invasion process.^14^^,^^15^ Despite extensive studies on animals and plants, the genetic basis of invasions by phytopathogens, particularly fungi and fungus-like taxa, remains largely underexplored.^16^^,^^17^

Another critical question in invasion biology is how invasive species rapidly adapt to novel climatic and environmental conditions.^18^ As invasive populations expand, they encounter abiotic and biotic factors that differ substantially from those in their native range. This imposes strong selective pressures that can sharply reduce fitness, especially in species with limited adaptive capacity.^19^^,^^20^ Although adaptive potential is generally associated with genomic diversity,^12^ reduced genetic diversity and bottlenecks in invasive species may limit their ability to adapt to new environments. This raises an important question: how do invasive species retain or recover adaptive potential despite reduced genomic diversity? The mechanisms enabling rapid adaptation under such constraints remain unclear, yet understanding them is essential for predicting and managing invasion outcomes.

Oomycetes are filamentous eukaryotic organisms that resemble fungi but are taxonomically distinct, belonging to the Stramenopila. They cause devastating diseases in diverse plant species, and represent a major global threat to crop production and food security.^21^ *Plasmopara viticola* is a biotrophic oomycete that causes one of the most destructive diseases, grapevine downy mildew worldwide.^22^ It produces sexual oospores that overwinter and germinate in spring, producing sporangia and zoospores for primary infection.^23^^,^^24^ Favorable mild temperatures and high humidity drive successive secondary infections, often resulting in epidemics.^24^

*Plasmopara viticola* is native to North America and was introduced to Europe in the 1870s, from where it rapidly spread worldwide.^6^ The North American lineage, *P. viticola* f. sp. *aestivalis*, is the primary cause of the global grapevine downy mildew pandemic.^6^^,^^25^ *Plasmopara viticola* was first reported in China in 1899 and is thought to have been introduced via a secondary wave of the worldwide spread from Europe.^6^ However, as a key center of origin for *Vitis*, China harbors abundant genetic resources in its wild grape populations, which has led to the hypothesis that China may represent an additional center of origin for *P. viticola*,^26^^,^^27^ although this hypothesis remains debated. Driven by the rapid expansion of grapevine cultivation, this pathogen is now found in most grape vineyards in China, including both temperate and subtropical zones with distinct climate conditions.^27^^,^^28^ Weather conditions have profound effects on the population biology of *P. viticola* and can influence the colonization strategies.^29^^,^^30^ These features make grape downy mildew in China a suitable model for studying the evolution and adaptation of invasive species following introduction into new environments.

This study aims to elucidate the invasion history and genomic basis of rapid adaptation in *P. viticola* in China, providing insights into the evolutionary mechanisms that enable persistence despite constrained genetic diversity. Specifically, we address a key ecological question: how invasive pathogens mitigate genetic depletion and achieve rapid climatic adaptation during range expansion. To this end, we performed whole-genome sequencing of 68 *P. viticola* samples collected from infected grapevine leaf lesions across four regions in temperate and subtropical China. These data were combined with 55 re-sequenced *P. viticola* isolates from Europe^23^^,^^31^ and published β-tubulin sequences from 390 *P. viticola* samples^6^ to reconstruct the origin and adaptive evolution of Chinese populations. This study provides evolutionary insights that are critical for understanding pathogen emergence and improving predictive disease management under climate change.

## Results

To investigate the origin, evolutionary dynamics, and climatic resilience of *P*. *viticola,* we combined whole-genome resequencing data from Chinese and European samples with published β-tubulin data including North American isolates. We collected downy mildew lesion samples from 19 Chinese vineyards across China, including three provinces (Liaoning, Yunnan, and Hunan) and the Beijing administrative region (Figures S1A and S1B). Among these, Beijing (BJ, 5 vineyards) and Liaoning (LN, 3) belong to the temperate region of northern China, whereas Yunnan (YN, 8) and Hunan (HN, 3) are located in subtropical region of southern China (Figure 1A).

**Figure 1:**
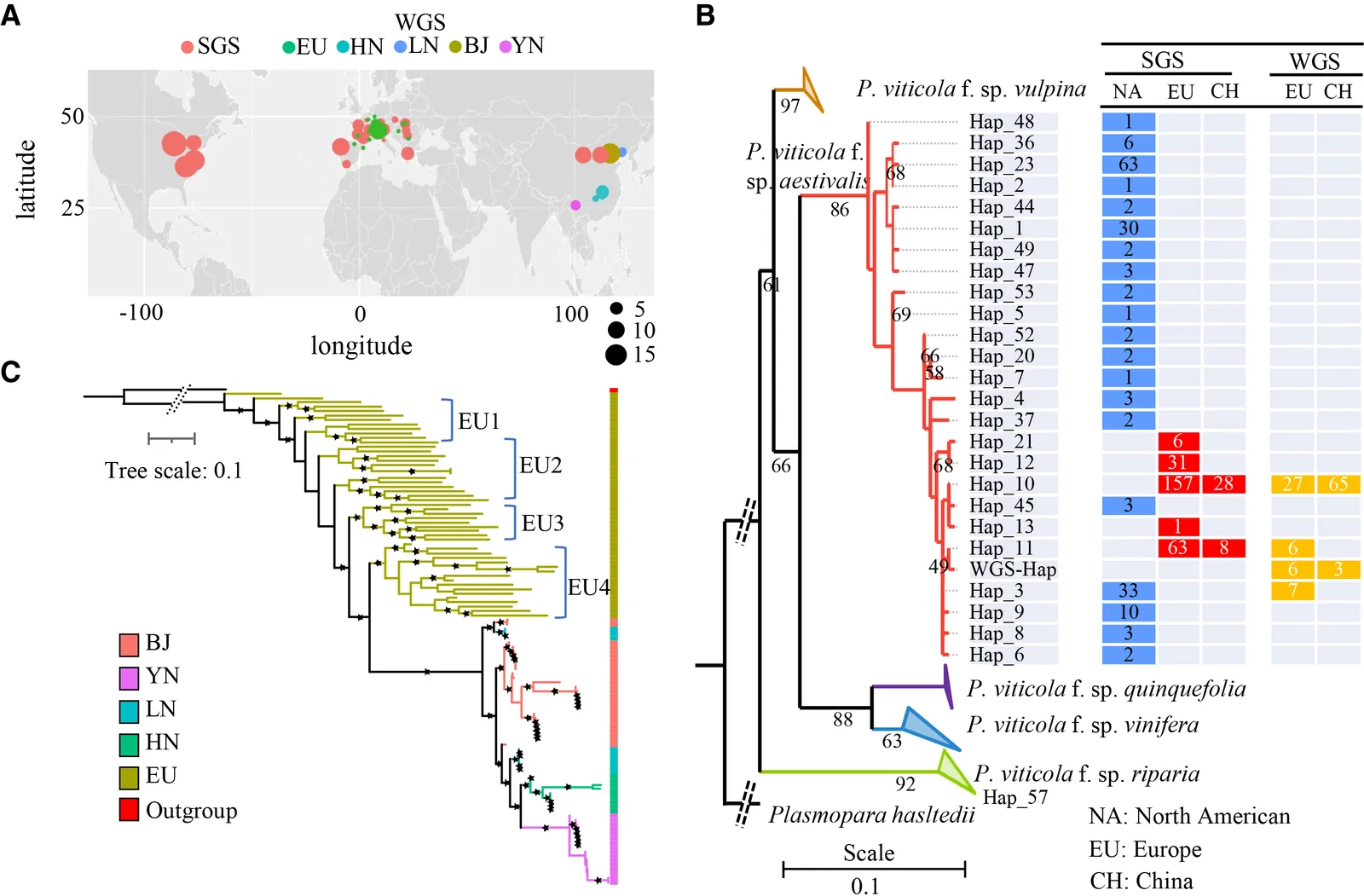
Phylogenetic analysis of *P. viticola* (A) Geographic distribution of *P. viticola* samples analyzed in this study. SGS: isolates from public data in Fontaine et al. based on β-tubulin DNA markers; WGS: whole-genome-sequenced samples from European vineyards (EU; literature sources) and Chinese vineyards in Beijing (BJ), Liaoning (LN), Hunan (HN), and Yunnan (YN). (B) Phylogeny based on β-tubulin sequences, integrating WGS and SGS data. β-tubulin loci were extracted from WGS assemblies using aTRAM, combined with SGS sequences, and used to construct a maximum-likelihood (ML) tree rooted with the *P. halstedii* tubulin gene as an outgroup. Formae speciales (f. sp.) are color-coded on branches, with haplotype frequencies shown in colored boxes indicating the total number of samples per haplotype from North America (NA), Europe (EU), and China (CH). Bootstrap support values are shown below branches. (C) Phylogeny of European and Chinese *P. viticola* samples based on WGS data. The ML tree was inferred from SNPs at 4-fold degenerate sites, rooted with *P. muralis*. Asterisks indicate internal nodes with bootstrap support >85%.

Genomic data from European vineyards were obtained from published sources.^23^^,^^31^ After mapping to the *P. viticola* PacBio reference genome^31^ and filtering for clones and low-quality samples, 26, 9, 9, 16, and 51 samples were retained for BJ, LN, HN, YN, and EU (Europe), respectively. Genome coverage and mapping rates varied substantially across samples (Table S1). Chinese populations showed lower pathogen mapping rates (mean: 34.2%) and higher host contamination (mean: 49.3%) compared to European populations (mean pathogen mapping: 76.7%; mean host mapping: 4.4%), likely reflecting variation in pathogen biomass and the higher proportion of host tissue in field-collected samples (Figures S1C and S1D).

Among the 60 Chinese samples, 56 were from *Vitis vinifera*, whereas four samples from HN were derived from the native wild grapevine *Vitis davidii* (Table S1). To compensate for the lack of whole-genome sequence (WGS) data for *P*. *viticola* samples from North America, we used the aTRAM software^32^ to assemble β-tubulin sequences from our WGS data and integrated these with previously published β-tubulin sequences from Fontaine et al.^6^

### North American ancestry and genetic relationships between Chinese and European *Plasmopara viticola*

To explore the origin of Chinese *P. viticola,* a total of 501 β-tubulin sequences from North America, Europe, and China, representing five *P. viticola formae speciales*, were analyzed. Using aTRAM,^32^ four distinct β-tubulin haplotypes were identified from the Chinese and European whole-genome sequencing datasets, two of which were shared between the two regions. In total, 51 haplotypes were detected among the 501 β-tubulin samples. Maximum-likelihood phylogenetic analysis revealed that all Chinese haplotypes clustered within *P. viticola* f. sp. *aestivalis*, a lineage native to North America (Figure 1B). Notably, the β-tubulin haplotypes detected in Chinese samples were also represented among European isolates. Thus, these data support the North American ancestral affiliation of the Chinese lineage but do not distinguish a direct North America-to-China introduction from a European-mediated transmission route.

We then used WGS data to investigate the relationship between Chinese and European *P. viticola* samples. By mapping reads to the *P. viticola* PacBio reference genome,^31^ we identified 1,549,647 high-confidence nuclear SNPs across 111 samples, including 75,758 fourfold-degenerate sites for phylogenetic analysis. The resulting phylogeny separated the Chinese and European samples into two major groups, with Chinese samples showing additional geographic substructure (Figure 1C).

Analysis of 49 mitochondrial SNPs similarly revealed substantial differentiation between most Chinese and European samples (Figure S2; Table S2). Most European samples retained the reference alleles, whereas Chinese samples predominantly carried alternative alleles at 41 mitochondrial positions. Phylogenetic analysis of mitochondrial SNPs similarly separated most Chinese and European samples (Figure S2B).

Notably, five samples assigned to the EU group, including the single sample from Georgia, carried mitochondrial alleles similar to those predominant in the Chinese populations, although their nuclear genomes clustered with other European samples in the nuclear phylogeny (Figure 1C). This mitonuclear discordance indicates historical allele sharing or retention of ancestral variation between the two lineages; therefore, gene flow and incomplete lineage sorting cannot be distinguished based on mitochondrial data alone.

### Population structure and demographic history of Chinese *P. viticola*

Multidimensional scaling (MDS) analysis based on Mash pairwise distances, coupled with ADMIXTURE clustering (K = 2), clearly separated Chinese and European *P. viticola* populations, confirming high divergence between them (Figures 2A and 2B; Figure S3). Cross-validation analysis indicated that K = 2 had the lowest CV error, at which Chinese and European samples formed two distinct ancestry components, indicating that geographic differentiation between China and Europe represents the dominant axis of population structure period.

**Figure 2:**
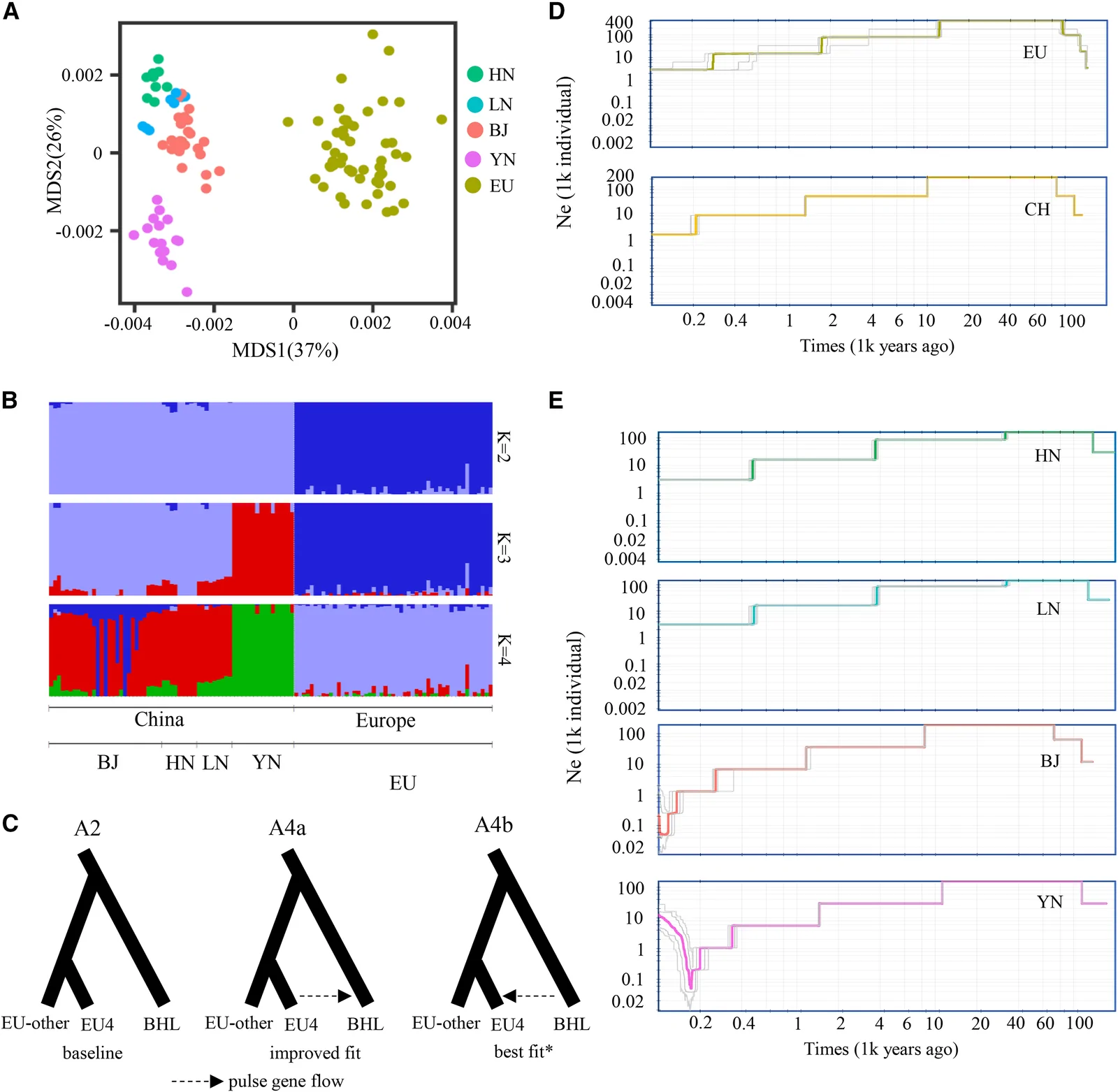
Population structure and demographic history of *P. viticola* (A) Multidimensional scaling (MDS) analysis of *P. viticola* samples based on the Mash genetic distance. (B) ADMIXTURE analysis of *P. viticola* based on sample genotype data. (C) Demographic model comparison among BHL, EU4, and EU-other using fastsimcoal2. A2 represents the best-fitting no-gene-flow model, whereas A4a and A4b extend A2 by incorporating a single pulse of post-divergence gene flow between EU4 and BHL in opposite directions. Both gene-flow models improved model fit relative to A2, with A4b yielding the lowest AIC. Dashed arrows indicate the forward-time direction of pulse gene flow. BHL: samples from BJ, HN, and LN. (D and E) Historical effective population size (*N*e) trajectories of *P. viticola* inferred using Stairway Plot 2 for the European and Chinese populations (D) and for the four Chinese subpopulations (E). YN showed the most pronounced recent reduction in *N*e, whereas BJ showed a weaker contraction. CH: all Chinese samples included in the whole-genome sequencing dataset.

Within China, BJ, LN, and HN showed relatively high genetic similarity, suggesting a broadly shared genetic background; these three populations were therefore grouped as BHL in subsequent demographic analyses. At K = 3, however, YN formed a distinct ancestry component relative to the other Chinese populations. This differentiation could reflect strong genetic drift associated with a population bottleneck, a distinct introduction history, or a combination of both processes.

Mitochondrial SNPs showed little differentiation among the Chinese populations (Figure S2A), consistent with shared or closely related maternal ancestry. However, mitochondrial similarity alone cannot distinguish between a single introduction followed by regional differentiation and multiple introductions from closely related source populations.

To further evaluate the demographic relationship between the Chinese and European populations, we compared alternative demographic models using fastsimcoal2 (Figure 2C; Figure S4; Table S3).^33^ Among three no-gene-flow topologies involving BHL, EU4 (a phylogenetically defined European subpopulation comprising primarily samples from Eastern Europe; Figure 1C), and the remaining European samples (EU-other), A2 provided the best fit, supporting the topology in which EU4 and EU-other share a more recent common demographic history relative to BHL.

We subsequently extended A2 by allowing a single post-divergence pulse of gene flow between EU4 and BHL in either direction. Both pulse-gene-flow models provided substantially better fits than the no-gene-flow A2 model, with A4b yielding the lowest AIC (Figure 2C; Table S3). These results support post-divergence allele exchange between the BHL and EU4-related lineages. Although the BHL-to-EU4 parameterization produced the best statistical fit, we interpret the inferred direction cautiously because demographic directionality may be sensitive to model optimization and unsampled population structure.

We separately evaluated three demographic scenarios for YN using BHL and EU4 as reference populations, with all models incorporating a YN bottleneck (Figure S4; Table S3). B2, in which YN and EU4 occupy the more closely related branches of the modeled demographic history, provided the best fit, whereas models grouping YN directly with BHL received substantially less support. These results are more consistent with a distinct introduction history for YN involving an EU4-related lineage, accompanied by a strong founder bottleneck, than with simple divergence of YN from the BHL population after a single introduction into China.

To further assess population size changes, we reconstructed historical effective population size trajectories using Stairway Plot 2.^34^ Both the combined Chinese and European populations showed substantial long-term changes in effective population size (Figure 2D). Among the Chinese subpopulations, YN exhibited the most pronounced recent reduction in effective population size, whereas BJ showed a weaker contraction and HN and LN lacked comparable recent declines (Figure 2E). These results demonstrate heterogeneous demographic histories among Chinese populations and provide independent evidence for a particularly strong founder effect or bottleneck in YN.

Together with the fastsimcoal2 model comparison, the YN bottleneck is more consistent with a distinct introduction history followed by strong founder effects than with genetic drift alone following simple derivation from BHL, although alternative demographic scenarios cannot be completely excluded.

### Reduced genetic diversity, regional differentiation, and restricted gene flow in Chinese *P. viticola*

Phylogenetic analysis of nuclear SNPs revealed pronounced geographic structure among Chinese *P. viticola* populations, with BJ, HN, and YN showing clear regional clustering. This pattern was also supported by multidimensional scaling (MDS) analysis. LN samples showed weaker geographic clustering, indicating greater genetic heterogeneity than the other Chinese populations. Together with the demographic analyses, these patterns indicate that regional population structure in China reflects a combination of founder effects, genetic drift, and potentially heterogeneous introduction histories. European samples were divided into four subpopulations (EU1–EU4) based on the nuclear phylogeny (Figure 1C). EU4 formed a relatively cohesive genetic cluster in the phylogeny, a pattern also supported by MDS and ADMIXTURE analyses (Figure S6A). Genetic diversity (π) analyses revealed significantly lower nucleotide diversity in the Chinese (CH) population than in the EU population (0.0074 vs. 0.0102, *p* < 0.001), consistent with substantial genetic loss associated with colonization and population establishment (Figure 3A). Among Chinese subpopulations, HN retained relatively high nucleotide diversity, whereas LN and BJ showed moderate reductions. YN exhibited the lowest nucleotide diversity (π = 0.0061, *p* < 0.01), consistent with the particularly strong founder bottleneck inferred from the demographic analyses (Figure 3A; Table S4). European subpopulations also showed modest differences in nucleotide diversity, indicating that demographic effects on diversity were not restricted to the Chinese populations.

**Figure 3:**
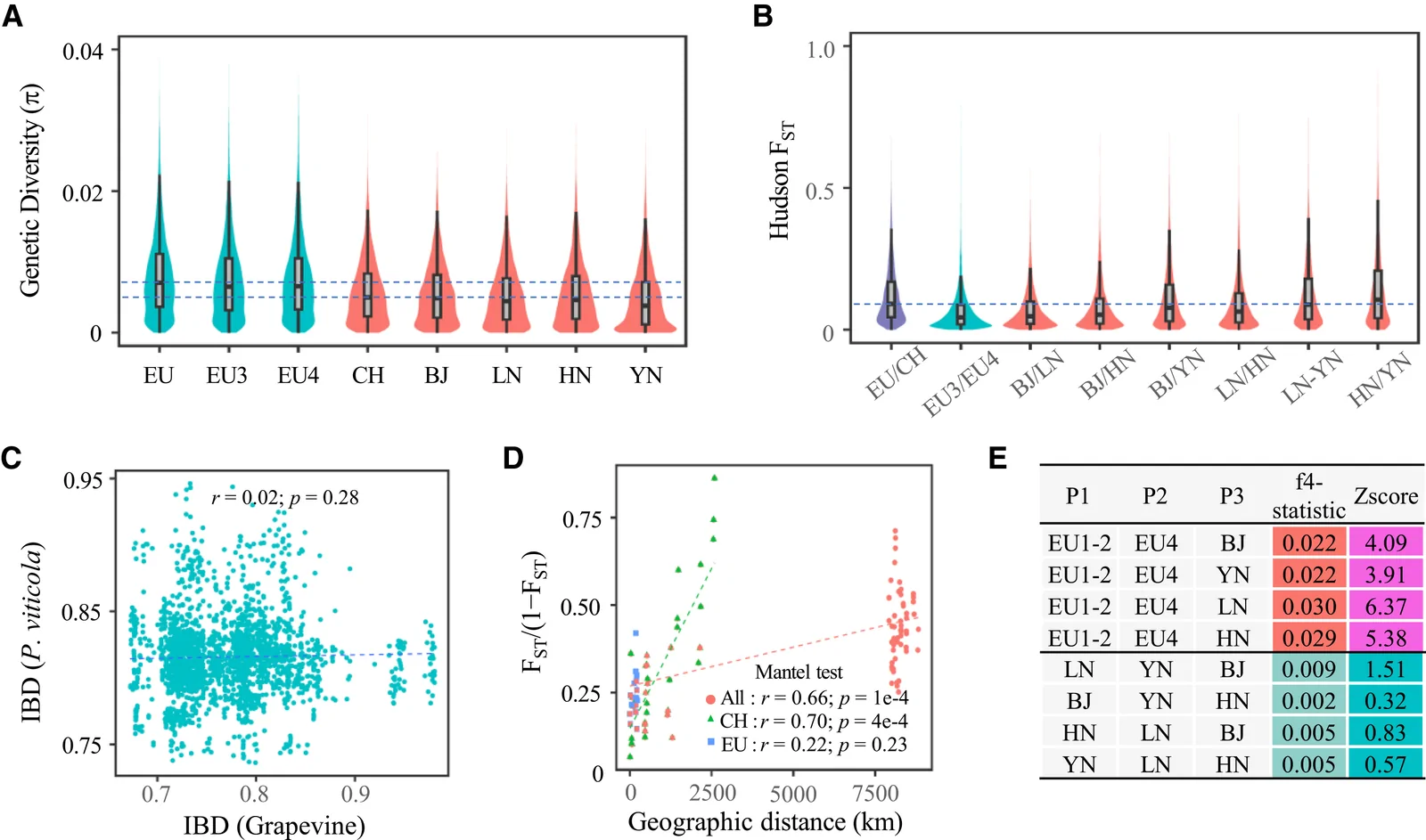
Population genetics of *P. viticola*: diversity, differentiation, host plasticity and introgression patterns (A) Genetic diversity (π) analysis across *P. viticola* populations. EU3 and EU4 denote EU subpopulations defined based on phylogenetic clustering observed in Figure 1C. The upper and lower dashed lines represent median π values for EU and CH, respectively. (B) Fixation index (F_ST_) analysis between and within EU and CH populations. The dashed horizontal line denotes the median F_ST_ between EU and CH. (C) Correlation analysis of pairwise identity by descent distance (IBD) between *P. viticola* and host grapevine. (D) Isolation-by-distance analyses in *P. viticola* populations assessed via Mantel tests at six European and nine Chinese geographic sites, as well as a combined dataset of 15 sites, each with at least three samples. (E) ABBA-BABA D-Statistic test for gene flow detection. Using the Dsuite software, potential introgression between the EU and CH subpopulations, as well as within the CH subpopulations were tested. EU1-2: the combination EU subpopulations of EU1 and EU2.

Genetic differentiation was assessed using the fixation index (F_ST_) between CH and EU populations, and among their respective subpopulations. The CH and EU differed significantly (F_ST_ = 0.136, Figure 3B). Within Europe, F_ST_ values among EU1–EU3 were low (0.054–0.067, Table S5), but increased when compared with EU4 (0.069–0.077), indicating greater divergence of this subpopulation. Within China, differentiation was more pronounced, with BJ, LN, and HN showing moderate divergence (0.082–0.108), and YN exhibiting the highest values (e.g., 0.170 vs. HN; Figure 3B; Table S5). These results demonstrate substantial regional population structure within China, particularly for YN.

The divergence observed among Chinese *P. viticola* populations may potentially be influenced by host genetic differences. Previous studies have shown that host genotypes can affect pathogen divergence.^35^ However, despite samples being collected from two different *Vitis* species, all HN samples cluster together in the phylogenetic tree, suggesting that host genotype is unlikely to be a major driver of population structure (Figure 1C). To further assess this, clean reads from 60 Chinese samples were mapped to the grapevine reference genome,^36^ yielding 6,180,502 SNPs. After linkage disequilibrium pruning using PLINK,^37^ pairwise identity-by-descent (IBD) distances revealed no significant correlation between host and pathogen genotypes (Figure 3C). These results suggest that host genetic variation has minimal influence on *P. viticola* population structure.

To assess whether geographic separation contributed to population differentiation, we tested for isolation by distance using samples from six European and nine Chinese sites (≥3 samples per site). Mantel tests revealed no significant correlation between genetic and geographic distance in Europe, but a significant positive correlation in China (*p* < 0.01; Figure 3D; Table S6). This pattern indicates stronger isolation by distance among Chinese populations, consistent with restricted long-distance dispersal during regional expansion.

Potential inter-regional gene flow was further evaluated using ABBA-BABA tests with *P. muralis* as an outgroup (|Z| > 2 for significance; Figure 3E; Table S3). No significant introgression signals were detected in the tested comparisons within China or within Europe. However, significant introgression signals were detected between EU4 and each of the Chinese subpopulations. This pattern is concordant with the fastsimcoal2 analyses, which favored demographic models incorporating historical relationships and post-divergence allele exchange between EU4-related and Chinese lineages. Together, these results indicate a more complex invasion history than a simple isolated introduction into China, although the available data do not uniquely identify the geographic direction or timing of these historical exchanges.

### Purifying selection purged putative deleterious genetic loads in Chinese *P*. *viticola*

Reduced genetic diversity may lead to decreased heterozygosity and allelic richness. Chinese *P. viticola* populations show reduced genetic diversity, retaining ∼90% of polymorphic sites compared with ∼99% in European populations. At the subpopulations level, European groups remained relatively stable (95–98% polymorphic sites), whereas Chinese subpopulations showed marked declines: BJ 85%, LN 80%, HN 78%, and YN just 71% (Figure S7A). This substantial loss of polymorphism, particularly in YN, may increase vulnerability to environmental stress and other selective pressures.

To evaluate the fitness consequences of reduced genetic diversity in Chinese *P. viticola*, we analyzed coding-region variants, which are more likely to affect gene function. Using ANNOVAR and SIFT4G,^38^^,^^39^ SNPs and indels were classified as synonymous (Syn), tolerated nonsynonymous (Tol, SIFT ≥ 0.05), putatively deleterious nonsynonymous (Del, SIFT < 0.05), or loss-of-function (LOF). Syn and Tol were treated as neutral, whereas Del and LOF mutations were considered deleterious. Derived alleles were inferred using *P. muralis* as an outgroup. Compared with EU populations, Chinese populations, particularly YN, showed increased fixation of derived Del and LOF mutations together with reduced polymorphism (Figure S7B).

Despite the high number of fixed homozygous deleterious mutations in Chinese populations, analysis of total derived LOF mutations (including both homozygous and heterozygous variants) suggested a higher degree of purging of highly deleterious LOF mutations in Chinese populations, particularly in YN, compared with European populations (Figure 4A). However, statistical analyses using Kruskal-Wallis tests followed by post-hoc pairwise comparisons did not detect significant differences among Chinese subpopulations for any mutation category. This indicates that, although variation is observable among subpopulations, these differences are not statistically supported. Although the total Del load did not differ significantly between Chinese and European populations, normalization to synonymous mutations revealed that Chinese subpopulations carried significantly lower relative Del loads (Figure 4A; Figure S8). These results suggest that, similar to animals and plants,^15^ plant pathogens may also purge deleterious mutations under genetic constraints.

**Figure 4:**
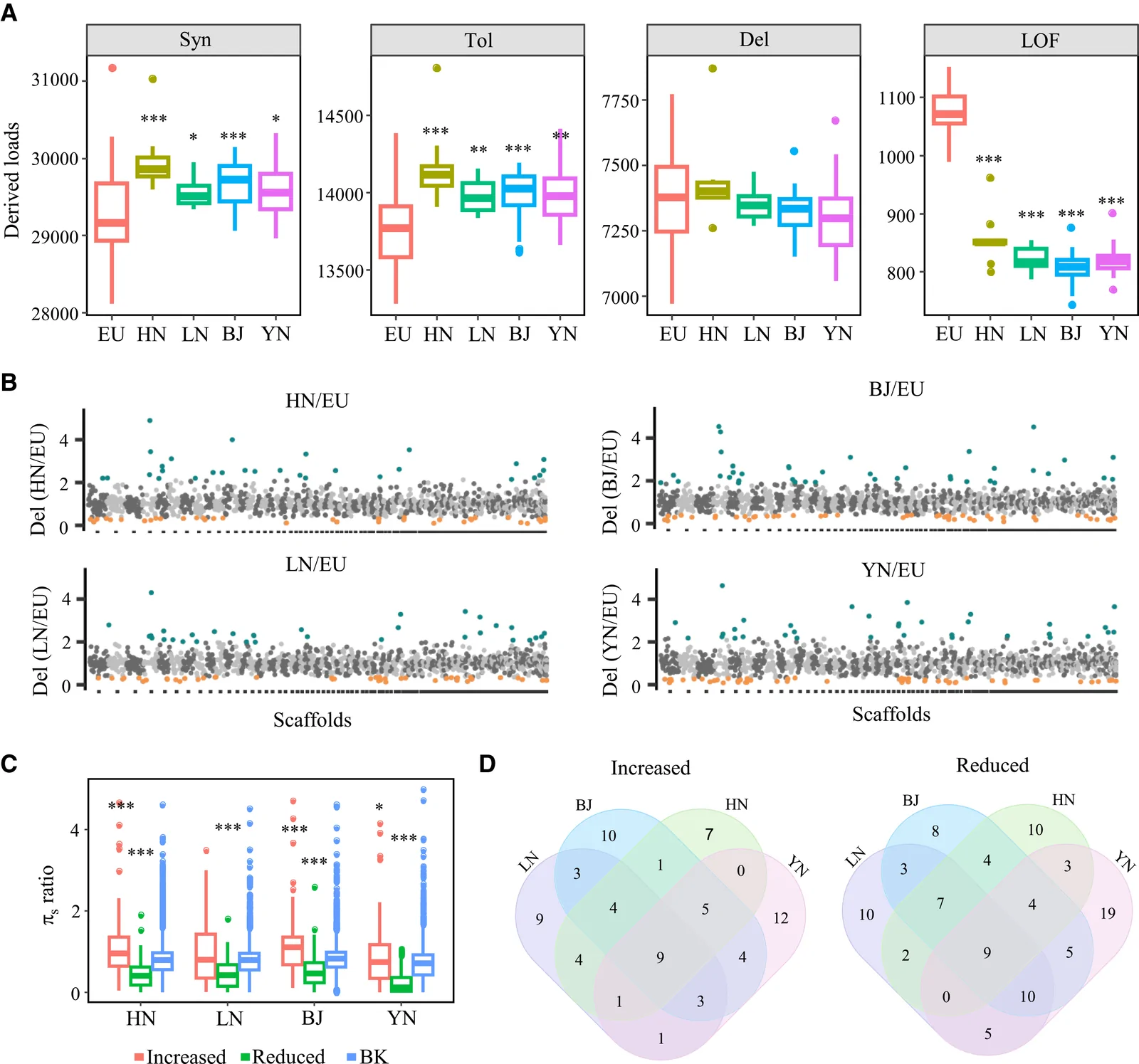
Purging of deleterious mutation loads in Chinese *P. viticola* (A) Derived mutation load in EU and CH populations across four mutation categories (Syn: synonymous mutation; Tol: tolerant missense mutation; Del: deleterious missense mutation; LOF: loss-of-function mutation). Each data point represents one isolate (EU, *n* = 51; HN, *n* = 9; LN, *n* = 9; BJ, *n* = 26; YN, *n* = 16). Boxplots show the median as the center line, the 25th and 75th percentiles as the lower and upper hinges, and whiskers extending to values within 1.5 × IQR; points beyond the whiskers are shown individually. Differences between European and Chinese populations were assessed using Wilcoxon rank-sum tests. Comparisons among Chinese subpopulations were evaluated using Kruskal-Wallis tests followed by post-hoc pairwise comparisons, which revealed no significant differences. (B) Genome-wide variation in derived Del mutation loads across Chinese populations. For each 50 kb non-overlapping genomic window across the genomes of Chinese populations, derived allele counts of deleterious mutations were normalized against those in EU samples. Blue dots: genomic regions with significantly higher deleterious mutation loads in Chinese *P. viticola* vs. EU samples. Orange dots: regions with reduced loads relative to EU samples. Significance thresholds (5th/95th percentile) were derived from permutation-based null distributions to account for stochastic variation (see STAR Methods). (C) Synonymous nucleotide diversity ratios (π_s_ ratios) in 50-kb genomic windows with significantly increased or reduced Del loads relative to EU. Each data point represents one 50-kb genomic window. The numbers of reduced-load windows were BJ, *n* = 50; LN, *n* = 46; HN, *n* = 39; YN, *n* = 55, and the numbers of increased-load windows were BJ, *n* = 39; LN, *n* = 34; HN, *n* = 31; YN, *n* = 35. BK denotes background windows without significant load differences. Increased- and reduced-load windows were separately compared with BK windows using Wilcoxon rank-sum tests. Boxplot elements are defined as in A. ∗∗∗: *p* < 0.001; ∗∗: *p* < 0.01; ∗: *p* < 0.05. (D) Venn diagram analysis of genomic regions in Chinese populations showing significantly elevated or reduced Del mutation loads in Chinese *P. viticola* vs. EU samples.

In small invasive populations, deleterious mutations may be removed by genetic drift or purifying selection.^15^ To assess the contribution of purifying selection, we examined synonymous diversity (π_s_) ratios between Chinese subpopulations and European samples. Lower π_s_ ratios indicate stronger purifying selection in Chinese subpopulations, whereas higher ratios suggest weaker selection in the same regions.^40^ To identify genomic regions with relatively low deleterious mutation loads, the *P. viticola* genome was partitioned into non-overlapping 50-kb windows. For each Chinese subpopulation, derived allele counts of Del and LOF mutations were scaled relative to European samples, serving as a proxy for genetic load. Using randomly selected genes as controls, we identified 50, 46, 39, and 55 of 50-kb windows in BJ, LN, HN, and YN, respectively, with significantly reduced Del loads compared with EU, and 39, 34, 31, and 35 windows with significantly increased Del loads (defined as values falling below the lowest 5% or above the highest 5% of the control distribution, Figure 4B). These regions are broadly dispersed across the genome without clear clustering. Regions with decreased Del loads exhibited significantly lower π_s_ ratios than the genomic backgrounds, indicating stronger purifying selection in Chinese subpopulations (Figure 4C). In contrast, regions with increased Del loads showed no consistent π_s_ pattern, suggesting more complex selection pressures.

Venn analyses revealed nine shared regions of decreased Del loads and nine increased Del loads across all Chinese subpopulations, suggesting that these patterns may have arisen prior to geographic divergence. Additionally, we identified 8, 10, 10, and 19 subpopulation-specific decreased-load windows in BJ, LN, HN, and YN, respectively (Figure 4D), indicating ongoing purifying selection following divergence. Genome-wide structural reorganization and localized removal of deleterious LOF mutations were also observed (Figure S9).

### Genomic signatures of thermal adaptation in Chinese *P*. *viticola*

To investigate how *P. viticola* adapts to diverse climatic conditions, we examined genomic differentiation between two geographically and climatically contrasting Chinese subpopulations: LN, from the highest latitude, and YN, from the lowest, which experience the lowest and highest mean annual temperatures, respectively (Figure 5A). Analysis of 19 WorldClim bioclimatic variables^41^ revealed pronounced differences between these two subpopulations in temperature-related variables (including bio1, bio3, bio6, bio9, and bio11), with LN showing the lowest and YN the highest values among all Chinese and European populations (Figure S10). In contrast, precipitation-related variables did not show extreme values in either population (Figure S11).

**Figure 5:**
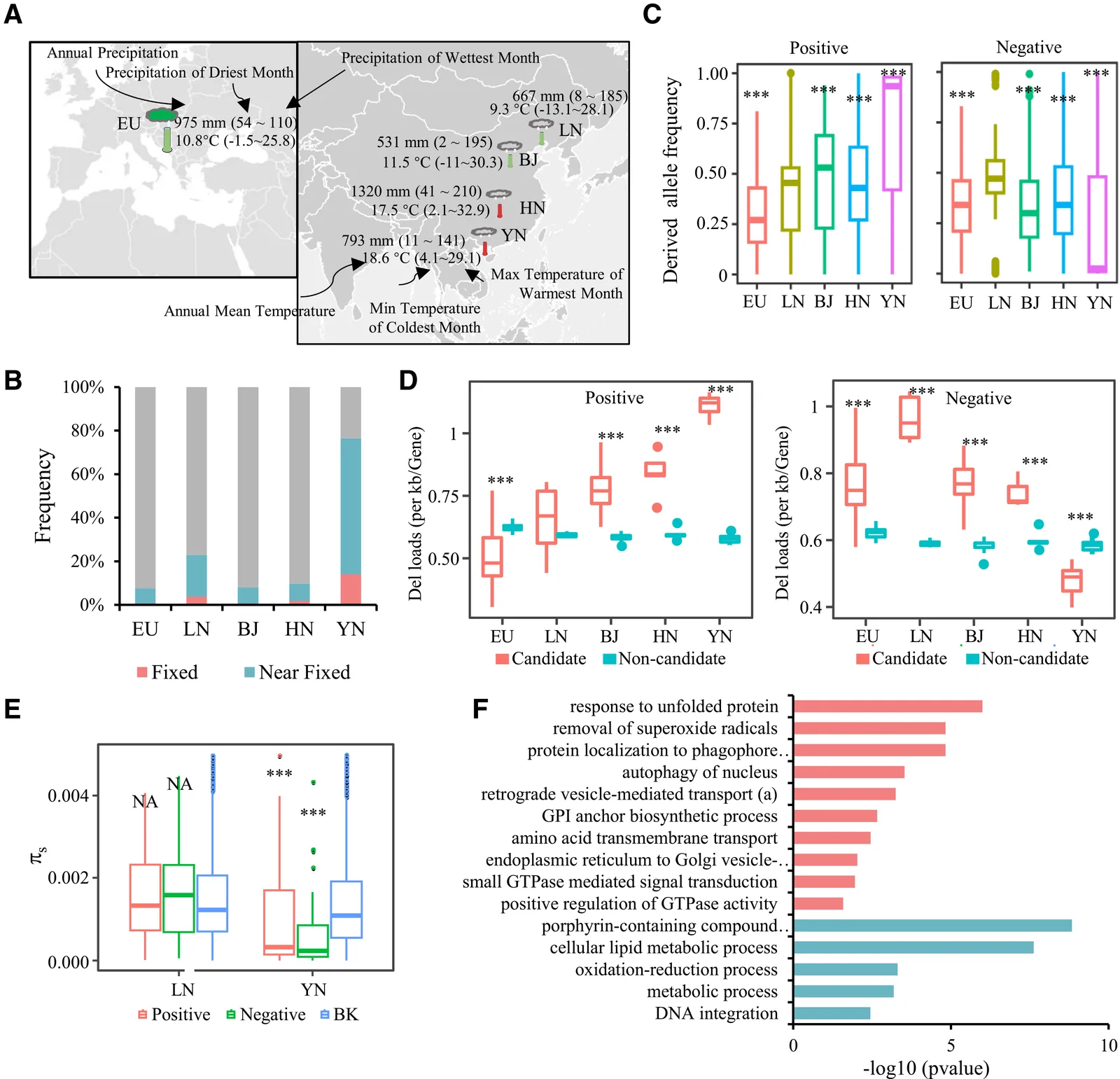
Analysis of high-divergence genomic regions in *P. viticola* LN and YN subpopulations (A) Representative bioclimatic variables across *P. viticola* populations. Values indicate regional averages for each bioclimatic factor (bio1 represents annual mean temperature). (B) Proportions of candidate adaptive SNPs with fixed alleles or nearly fixed alleles (0.9 ≤ major allele frequency < 1) across different populations. Only candidate adaptive SNPs with distinguishable derived/ancestral alleles are included. (C) Distribution of derived allele frequencies of candidate adaptive SNPs across *P. viticola* populations. Each data point represents one candidate SNP. Candidate SNPs were classified as positively or negatively associated with bio1 based on the correlation between derived allele frequency and annual mean temperature. The positive and negative groups contained *n* = 449 and *n* = 677 SNPs, respectively. Boxplots show the median, 25th–75th percentiles, and 1.5 × IQR whiskers. Differences between LN and the other populations were assessed using Wilcoxon rank-sum tests. (D) Del loads calculated for candidate adaptive and non-candidate gene sets across *P. viticola* populations. Candidate genes were classified as positively or negatively associated with bio1 according to the most highly differentiated SNP within each gene. Each data point represents one isolate (EU, *n* = 51; LN, *n* = 9; BJ, *n* = 26; HN, *n* = 9; YN, *n* = 16). Boxplot elements are defined as previous. Candidate and non-candidate genes within each population were compared using Wilcoxon rank-sum tests. (E) Local synonymous nucleotide diversity associated with candidate adaptive genes in the LN and YN populations. Synonymous nucleotide diversity (π_s_) was calculated in non-overlapping 5-kb genomic windows. Windows containing positively associated (positive), negatively associated (negative), or non-candidate background genes (BK) were grouped accordingly. Each data point represents one unique 5-kb genomic window. (Positive, *n* = 62; negative, *n* = 57; BK, *n* = 5,051). Boxplot elements are defined as above. Positive and negative groups were separately compared with BK using Wilcoxon rank-sum tests. ∗∗∗: *p* < 0.001; ∗∗: *p* < 0.01; ∗: *p* < 0.05. (F) GO enrichment analysis of candidate adaptive genes with distinguishable derived/ancestral alleles (soft red) versus those without (desaturated cyan).

We identified highly differentiated genomic regions between LN and YN using the top 5% F_ST_ values and detected putative selective sweeps based on the lowest 5% Tajima’s D, defining 356 outlier regions of 5 kb each. Within these, 12,913 highly differentiated SNPs were found (top 5% F_ST_), including 3,081 within genes. Environmental association analysis of gene-associated SNPs across EU and four Chinese subpopulations was conducted using 19 bioclimatic variables. This analysis identified 2,290 candidate SNPs that were significantly correlated (Spearman correlation, *p* < 0.1) with at least one bioclimatic variable. Of these, 1,126 candidates showed clearly distinguishable ancestral and derived states using *P. muralis* as the outgroup. Temperature-related variables, particularly bio1 (annual mean temperature), showed the strongest associations (Figure S12), suggesting that temperature is a major factor associated with allele frequency divergence between LN and YN.

Analysis of candidate SNPs revealed that nearly 80% exhibited major alleles that were fixed or nearly fixed (allele frequency 90%–100%) in the YN population, whereas in European and other Chinese subpopulations, no more than 20% of the major alleles reached such high frequencies. This pattern was consistent across all candidate SNPs (Figure S13A), including those with defined ancestral or derived states (Figure 5B) and deleterious variants (Figure S13B). Assuming that allele fixation reflects the selection intensity, these results suggest that *P. viticola* populations in warmer environments (e.g., YN) may experience stronger selective pressures than those in cooler climates (e.g., LN).

We next analyzed 1,126 candidate SNPs located within 187 genes to investigate the genomic basis of temperature adaptation across *P. viticola* populations. Genes were categorized based on their associations with bio1 into two groups: positively correlated (derived allele frequency increases with temperature) and negatively correlated (derived allele frequency decreases with temperature). Given the higher bio1 values in YN, derived alleles tended to accumulate in positively correlated genes and decrease in negatively correlated genes (Figure 5C).

For positively correlated genes, EU samples exhibited lower Del but relatively higher LOF loads than their genomic background, whereas all Chinese subpopulations showed significantly elevated Del and LOF loads, suggesting a relative relaxation of purifying selection (Figure 5D; Figure S14A). The YN populations exhibited the highest Del and LOF burdens, with markedly reduced π_s_ values compared with the genomic background—a pattern not observed in other Chinese or European samples—suggesting that some of these variants may be conditionally advantageous and under positive selection in high-temperature environments (Figure 5E; Figure S15).

For negatively correlated genes, EU and most Chinese subpopulations exhibited elevated Del but similar or relatively lower LOF loads compared with their genomic background. In contrast, the YN populations carried minimal Del and LOF mutations (Figure 5D; Figure S14B), together with markedly reduced π_s_ values (Figure 5E; Figure S15), indicating strong purifying selection acting to efficiently remove deleterious alleles.

### Functional analysis of thermal adaptation-associated genes

Among 187 genes containing SNPs with distinguishable ancestral and derived states, Gene Ontology (GO) enrichment analysis identified 10 significantly enriched biological processes (FDR <0.05) (Figure 5F). The most prominent category was response to unfolded protein (RUP), which is critical for cellular protein-folding quality control.^42^ Elevated temperatures can disrupt protein folding and lead to the accumulation of cytotoxic misfolded proteins; thus, selection acting on RUP-related genes may contribute to thermal stress tolerance in *P. viticola*.^43^^,^^44^ Other enriched pathways included superoxide removal, nuclear autophagy, and Golgi-ER vesicle transport (Figure 5F), all of which are involved in maintaining protein homeostasis and mitigating heat-induced oxidative stress.^45^^,^^46^^,^^47^^,^^48^

For the 53 genes containing SNPs with uncertain ancestral state, GO analysis revealed significant enrichment in pathways related to lipid metabolism, oxidation-reduction reactions, DNA integration, and other stress-associated functions (Figure 5F). Temperature fluctuations can influence membrane fluidity, disrupt redox homeostasis through ROS accumulation, and compromise genome stability,^49^^,^^50^ suggesting that these processes may be involved in adaptive responses to environmental stress.

We identified *upl6* (PVIT_0002541), an E3 ubiquitin ligase gene, containing 15 candidate SNPs (10 Syn, 2 Tol, 2 Del, and 1 intronic). E3 ligases play key roles in ubiquitination and proteasomal degradation, which are essential for pathogen responses to environmental stress.^51^^,^^52^ In our analysis, the major allele frequencies of these SNPs exceeded 97% in YN, whereas in other populations they ranged from 53% to 76% (Figure 6A). The two Del alleles in YN were rare (allele frequencies <2%). In addition, nucleotide diversity calculated across all SNPs within *UPL6* was markedly reduced in YN (Figure 6B). Together, these patterns are consistent with strong purifying selection at this locus, potentially contributing to the maintenance of UPL6 function and stress tolerance.

**Figure 6:**
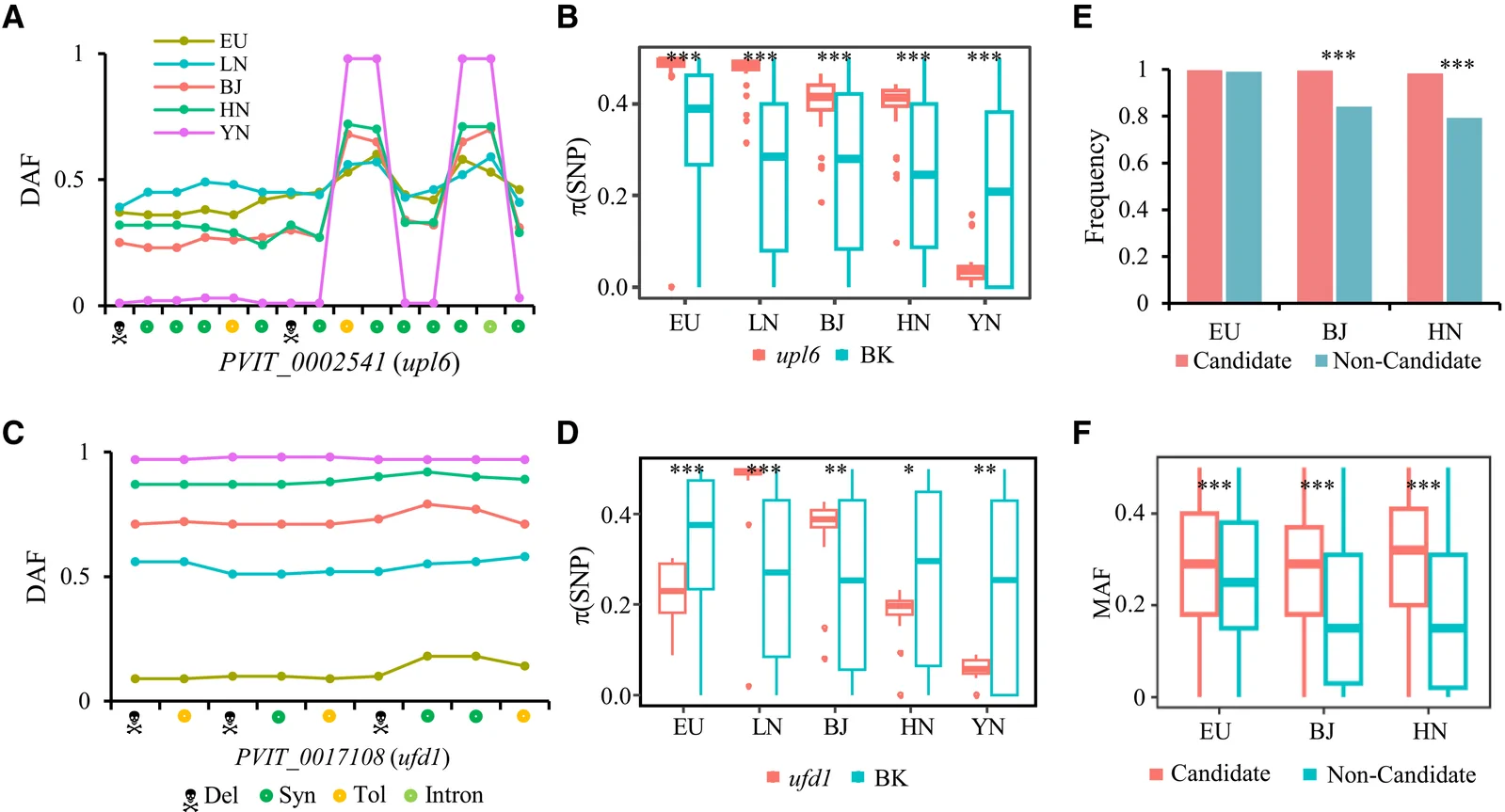
Investigation of candidate adaptive genes and characterization of standing genetic variation in candidate adaptive SNPs (A) Derived allele frequency (DAF) of 15 candidate adaptive SNPs in an E3 ubiquitin-protein ligase gene *upl6* (PVIT_0002541) across *P. viticola* populations. (B) Genetic diversity of SNPs within *upl6* (PVIT_0002541) compared with the genomic background (BK) across *P. viticola* populations. Each data point represents the nucleotide diversity (π) calculated for one SNP. The *upl6* group included *n* = 51 SNPs, and BK comprised 10,000 randomly selected SNPs from gene regions across the genomic background. Differences between *upl6* and BK were assessed using Wilcoxon rank-sum tests. (C) DAF of candidate adaptive SNPs in the ubiquitin-recognition adapter protein gene *ufd1* (PVIT_0017108) across *P. viticola* populations. (D) Genetic diversity of SNPs within *ufd1* (PVIT_0017108) compared with the genomic background (BK) across *P. viticola* populations. Each data point represents the nucleotide diversity (π) calculated for one SNP. The *ufd1* group included *n* = 30 SNPs, and BK comprised 10,000 randomly selected SNPs from gene regions across the genomic background. Differences between *ufd1* and BK were assessed using Wilcoxon rank-sum tests. (E) Proportions of polymorphic sites among candidate adaptive and non-candidate SNPs in EU, BJ, and HN populations. The candidate set comprised 2,290 SNPs, and 10,000 non-candidate SNPs were randomly selected as the background set. Differences in polymorphic-site frequencies between the two groups were assessed using χ^2^ tests. (F) Minor allele frequency (MAF) distributions of the same candidate and non-candidate SNPs in EU, BJ, and HN populations. Each data point represents one SNP. Differences between candidate and non-candidate SNPs within each population were assessed using Wilcoxon rank-sum tests. For all boxplots, the center line indicates the median, boxes represent the 25th–75th percentiles, and whiskers extend to values within 1.5 × the interquartile range (IQR). Points beyond the whiskers are shown individually. ∗∗∗: *p* < 0.001; ∗∗: *p* < 0.01; ∗: *p* < 0.05.

UFD1 (PVIT_0017108), an endoplasmic reticulum-associated degradation pathway adapter, harbored 9 SNPs (3 Syn, 3 Tol, and 3 Del). This protein facilitates the export of misfolded ER proteins for degradation via the ubiquitin-proteasome system (UPS).^53^^,^^54^ Chinese populations showed significantly higher derived allele frequencies than EU (Figure 6C), indicating pronounced lineage differentiation at this locus. In YN, variants were nearly fixed (MAF >97%) accompanied by low π, together with a high frequency of nonsynonymous mutations, suggesting reduced diversity potentially driven by selection (Figure 6D). The persistence of deleterious variants may reflect conditional effects under specific environmental contexts.

### The predominant role of standing genetic variation in thermal adaptation

The rapid spread of invasive pathogens suggests founder events and reduced genetic diversity do not necessarily constrain adaptation. We next investigated how natural selection acts on genomic variation to facilitate rapid adaptation. Natural selection can act on pre-existing genetic variation or on mutations that arise after population establishment.^55^^,^^56^ To evaluate the contribution of pre-existing variation to climatic differentiation, we examined 2,290 differentiated SNPs between the LN and YN populations and assessed the occurrence and allele frequencies of these variants in other populations. Variants that remain polymorphic across geographically distinct populations are unlikely to represent mutations arising uniquely during recent differentiation of LN or YN and are therefore consistent with selection acting on pre-existing variation.

Candidate and non-candidate SNPs showed markedly different polymorphism patterns. In EU, 99.6% of candidate SNPs were polymorphic, compared with 99.1% of non-candidate SNPs (Figure 6E). The difference was substantially greater in Chinese populations: 99.5% (BJ) and 98.2% (HN) of candidate SNPs remained polymorphic, whereas only 84.1% (BJ) and 79.2% (HN) of non-candidate SNPs were polymorphic (χ^2^ test, *p* < 0.001). The widespread retention of candidate variants across multiple populations indicates that many of the alleles associated with LN-YN differentiation were already present outside the focal populations, supporting selection on pre-existing variation rather than exclusively on population-specific new mutations.^56^

Candidate SNPs also showed significantly higher minor allele frequencies than non-candidate SNPs in EU, BJ, and HN (Wilcoxon rank-sum test, *p* < 0.001; Figure 6F). In addition, 155 candidate SNPs annotated as Del or LOF showed similarly elevated polymorphic frequencies and minor allele frequencies relative to non-candidate variants (*p* < 0.001; Figure S16). These results further suggest that variants maintained at appreciable frequencies in the broader gene pool provided genetic substrates upon which subsequent environmental selection could act.

## Discussion

Earlier studies based on a limited number of loci and geographically restricted sampling proposed conflicting hypotheses for the origin of Chinese *P. viticola*, including direct introduction from North America, introduction via Europe, and mixtures of endemic and introduced lineages.^6^^,^^27^^,^^57^ By combining β-tubulin data from North America, Europe, and China with whole-genome data from Chinese and European populations, our results provide strong support for a North American ancestral origin of the lineage represented by Chinese *P. viticola*. However, because North American populations were not included in our whole-genome dataset, the immediate geographic route by which this lineage reached China cannot be resolved directly. The sharing of β-tubulin haplotypes between Chinese and European isolates is consistent with this distinction, because this marker identifies their broader affiliation with the North America-native f. sp. *aestivalis* lineage but does not resolve the immediate source population of the Chinese invasion.

Genome-wide analyses revealed strong differentiation between contemporary Chinese and European populations, whereas both ABBA-BABA tests and demographic model comparisons indicated historical connections between EU4-related and Chinese lineages. In particular, models incorporating post-divergence allele exchange between EU4 and BHL provided substantially better fits than no-gene-flow models. In addition, the best-supported model for YN was more consistent with a distinct introduction history involving an EU4-related lineage together with a strong founder bottleneck than with simple derivation of YN from BHL. Together, these results suggest that the invasion history of Chinese *P. viticola* was more complex than a single isolated introduction, involving historical connections with European-related lineages and potentially heterogeneous introduction histories among Chinese populations. This pattern is compatible with a European bridgehead scenario proposed by Fontaine et al.^6^ although the available data do not uniquely resolve the geographic direction or timing of these historical exchanges.

Exotic invaders often experience genetic depletion during colonization,^5^ although repeated introductions and gene flow can partially alleviate such losses.^11^^,^^13^ Despite evidence for a more complex introduction history, Chinese *P. viticola* populations still showed substantially reduced genomic diversity relative to European populations, indicating that strong founder effects accompanied their establishment in China. The magnitude of this reduction varied among regions, with YN showing the strongest loss of diversity and the most pronounced demographic bottleneck. Our results therefore suggest that a complex introduction history does not necessarily preclude severe founder effects. Similar reductions in genetic diversity have also been reported during the global spread of *P. viticola*,^6^ suggesting that demographic bottlenecks are a recurrent feature of its invasion history.

The decrease of genetic diversity in founder populations may generate a high deleterious load in *P. viticola*, which may result in inbreeding depression and a potential reduction in fitness.^58^ Notably, our analysis reveals a purging process of deleterious alleles in Chinese populations relative to the European population, a phenomenon corroborated by studies on bottlenecked animal and plant populations.^59^^,^^60^^,^^61^^,^^62^ Deleterious mutations may have a negative effect on fitness and can cause lower lifetime reproductive rates and be more frequently exposed to selection in the homozygous state.^63^ These findings suggest that purging of deleterious mutations may help mitigate the fitness consequences of genetic bottlenecks in *P. viticola*.

*Plasmopara viticola* occurs worldwide, from temperate to tropical vineyards,^6^^,^^29^ and its spread with grapevine cultivation has likely imposed strong climatic selection pressures.^16^ In China, the pathogen has successfully colonized contrasting environments, including cooler high-latitude region represented by LN and the warmer low-latitude region represented by YN. Although gene flow can potentially introduce adaptive alleles into newly established populations,^64^ we detected no significant introgression signals among the tested Chinese population pairs, while isolation-by-distance analyses indicated restricted long-distance dispersal. These observations suggest that the strong climatic differentiation observed among Chinese populations is unlikely to be explained primarily by recent inter-regional introgression. Host genetic variation also showed little association with pathogen population structure (Figure 3C), further supporting an important role for environmental selection.

Comparative genomic scans reveal contrasting selective patterns between LN and YN, consistent with their markedly different thermal environments. In LN, we detected signals predominantly associated with purifying selection, with limited evidence for strong positive selection (Figures 4 and 5). In contrast, YN showed stronger population-specific selective signals, including both the removal of putatively deleterious variants and positive selection in genomic regions containing variants potentially associated with heat tolerance (Figure 5). Genes within these regions were enriched for functions related to thermal stress responses, including proteostasis, membrane-associated processes, and antioxidant defenses.^45^^,^^46^^,^^49^^,^^50^ These results suggest that populations occupying contrasting climatic environments have experienced different selective regimes following their establishment in China.

Our findings support an important role for standing genetic variation in the climatic differentiation of invasive *P. viticola* populations.^65^ Candidate variants associated with LN-YN differentiation were frequently retained as polymorphisms in other populations and generally occurred at higher allele frequencies than non-candidate variants (Figures 6E and 6F).This distribution is more consistent with selection acting on pre-existing variation than with an exclusively *de novo* origin of adaptive alleles after regional population establishment.^56^ Although our data cannot exclude contributions from newly arisen mutations, standing variation would provide an immediately available source of genetic diversity upon which selection could act during colonization of contrasting environments.^55^^,^^66^ Importantly, this process occurred despite substantial founder effects in Chinese populations. YN, which showed the strongest demographic bottleneck and a distinct introduction history, nevertheless retained candidate variants upon which selection could act. Because many of these candidate variants were also polymorphic in other populations, their interpretation as pre-existing variation does not depend on an assumed recent divergence between YN and BHL. Thus, even under strong demographic constraints, a subset of pre-existing variants can persist and subsequently change in frequency under environmental selection.

These results contribute novel insights into pathogen adaptation mechanisms under genetic constraint. They highlight how natural selection can reconfigure deleterious mutation landscapes to address environmental challenges and reveal the strategic importance of standing genetic resources in enabling rapid invasive success. This mechanistic framework could inform predictive models of disease emergence in novel environments, particularly under climate change scenarios where pathogens face unprecedented temperature shifts.

### Limitations of the study

Despite the insights provided by this study, several limitations should be acknowledged. First, the geographic representation of our whole-genome resequencing dataset is incomplete. Our sampling is largely confined to China and Europe, with no inclusion of populations from North America or other regions where the pathogen is prevalent. Consequently, our data are insufficient to fully resolve global population structure, and do not allow direct inference of the relationships among Chinese, European, and North American populations. Second, within China, the number of sampling locations remains limited. This restricted spatial coverage constrains our ability to precisely reconstruct the transmission routes and dispersal dynamics of the pathogen across different regions. Third, some populations are represented by relatively small sample sizes, which may reduce the statistical power of population genetic analyses and potentially affect the resolution of inferred patterns. Despite these limitations, the consistency of signals observed across multiple analyses supports the robustness of our main conclusions regarding population differentiation and evolutionary patterns. Future studies incorporating broader geographic sampling—particularly from currently underrepresented regions such as North America—as well as denser sampling within China, will be essential not only for refining the global population structure and transmission history of this pathogen, but also for advancing our understanding of the adaptive mechanisms underlying its spread across diverse environments.

## Resource availability

### Lead contact

Requests for further information and resources should be directed to and will be fulfilled by the lead contact, Jiye Yan (jiyeyan@vip.163.com).

### Materials availability

This study did not generate new or unique reagents.

### Data and code availability

• Raw genome sequence reads have been deposited in the Genome Sequence Archive (GSA) under accession GSA: CRA020626. The data are publicly accessible at https://ngdc.cncb.ac.cn/gsa.
• Previously published whole-genome resequencing datasets analyzed in this study are available from NCBI under accessions NCBI: PRJNA644583 and NCBI: PRJNA329579. The *Plasmopara muralis* dataset used for outgroup analyses is available under NCBI: PRJNA448661. Published β-tubulin sequences are available from GenBank under accessions GenBank: MW053069-MW053145, and the *P. viticola* PacBio reference genome is available under accession NCBI: GCA_001695595.3.
• This paper does not report original code.
• Any additional information required to reanalyze the data reported in this paper is available from the lead contact upon request.

## Acknowledgments

We thank Prof. Changfu Tian for insightful discussions and critical feedback on the manuscript. This work was supported by the 10.13039/501100007934BAAFS Fund for Distinguished Research Teams (Grant No. JKTD2025002).

## Author contributions

J.Y., X.L., and X.W. conceived the project. X.W. and J.Y. designed the project. X.W., Y.L., J.P., W.Z., Q.X., H.W., M.L., Y.Z., and L.W. collected pathogen samples. X.W., Y.L., J.P., and J.X. conducted data analyses. X.W., I.M., Y.L., J.P., J.X., and J.Y. prepared the article. All authors read and approved the final manuscript.

## Declaration of interests

The authors declare no competing interests.

## Declaration of generative AI and AI-assisted technologies in the manuscript preparation process

Generative AI tools were used to assist with language editing during manuscript preparation. The authors critically reviewed and revised the manuscript and are fully responsible for its content.

## STAR★Methods

### Key resources table

**Table:** 

REAGENT or RESOURCE	SOURCE	IDENTIFIER
**Deposited data**		

Whole-genome resequencing data of Chinese *P. viticola* generated in this study	This paper	GSA: CRA020626
Published whole-genome resequencing data of 54 European *P. viticola*	Dussert et al.^23^	NCBI: PRJNA644583
Published whole-genome resequencing data of European *P. viticola* PV221	Dussert et al.^31^	NCBI: PRJNA329579
Published whole-genome resequencing data of *P. muralis*	Dussert et al.^31^	NCBI: PRJNA448661
β-tubulin sequences of *P. viticola*	Fontaine et al.^6^	GenBank: MW053069-MW053145
PacBio reference genome of *P. viticola*	Dussert et al.^31^	NCBI: GCA_001695595.3

**Software and algorithms**		

fastP v0.23.4	Chen et al.^67^	https://github.com/OpenGene/fastp
BWA-MEM v0.7.17	Li and Durbin.^68^	https://github.com/lh3/bwa
SAMtools v1.10	Li et al.^69^	https://github.com/samtools/samtools
GATK v4.1.8.0	McKenna et al.^70^	https://github.com/broadinstitute/gatk
VCFtools v0.1.17	Danecek et al.^71^	https://github.com/vcftools/vcftools
PLINK v1.90	Purcell et al.^37^	https://www.cog-genomics.org/plink
Mash v2.3	Ondov et al.^72^	https://github.com/marbl/Mash/releases
ape v5.8-1	Paradis and Schliep.^73^	https://cran.r-project.org/web/packages/ape/index.html
ADMIXTURE v1.3	Alexander et al.^74^	https://dalexander.github.io/admixture/
Dsuite v0.5 r52	Malinsky et al.^75^	https://github.com/millanek/Dsuite
aTRAM v2.4.3	Allen et al.^32^	https://github.com/juliema/aTRAM
MAFFT v7.453	Katoh and Standle.^76^	https://mafft.cbrc.jp/alignment/software/
IQ-TREE2 v2.4.0	Minh et al.^77^	https://github.com/iqtree/iqtree2
Stairway Plot 2 v2.1.2	Liu and Fu.^34^	https://github.com/xiaoming-liu/stairway-plot-v2
fastsimcoal2 v2.7.0	Excoffier et al.^33^	https://cmpg.unibe.ch/software/fastsimcoal2/
ANNOVAR (2018Apr16)	Wang et al.^38^	https://annovar.openbioinformatics.org/en/latest/
SIFT4G v6.0	Vaser et al.^39^	https://github.com/rvaser/sift4g

**Other**		

WorldClim v2	Fick and Hijmans.^41^	https://worldclim.org/

### Experimental model and study participant details

#### Sample collection

During the 2020 growing season, grapevine leaves showing typical downy mildew symptoms were collected from 19 vineyards across four regions in China. For each infected plant, 2–3 symptomatic leaves were sampled depending on disease severity (two leaves for heavily infected plants with abundant sporulation and three leaves for plants with less visible symptoms). In total, 27 samples were collected from subtropical regions (Yunnan and Hunan) and 41 from temperate regions (Beijing and Liaoning) (Figure 1A; Table S1).

Lesions were defined as areas exhibiting visible chlorotic or necrotic patches on the adaxial surface and characteristic white, downy sporulation on the abaxial surface. The presence of sporangia was assessed visually based on the appearance of abundant downy sporulation. Lesion tissues bearing visible sporangia were excised using sterilized scissors, placed individually into sterile tubes, and stored at −80 °C until DNA extraction (Figures S1A and 1B).

### Method details

#### Whole-genome sequencing, read mapping, and variant calling

Genomic DNA was extracted from lesion tissues using a modified cetyltrimethylammonium bromide (CTAB) method. Briefly, lesion tissues with visible sporulation were ground in liquid nitrogen, followed by incubation in CTAB extraction buffer. DNA was purified by chloroform–isoamyl alcohol extraction and precipitated with isopropanol. The resulting DNA was washed with ethanol, air-dried, and resuspended in TE buffer. To minimize host DNA contamination, DNA extraction was performed on lesion tissues enriched with pathogen sporulation (Figure S1B).

Genomic libraries were prepared with the NEB Next® Ultra^TM^ DNA Library Preparation Kit (Ipswich, MA, USA) and sequenced on an Illumina NovaSeq 6000 platform by Novogene Bioinformatics Technology Co., Ltd. (Beijing, China), generating an average of 43 million 150-bp read pairs per sample. After quality filtering with fastp,^67^ the proportion of retained clean reads exceeded 99% in Chinese samples. Cleaned reads were mapped to the *P. viticola* PacBio reference genome using BWA-MEM v.0.7.17,^31^^,^^68^ and reads with mapping scores less than 20 were excluded to ensure high-confidence alignments.

Mapping statistics, including the proportions of reads aligned to the host (*Vitis vinifera*) and *P. viticola*, are summarized in Table S1. No explicit filtering based on host contamination thresholds was applied, as downstream analyses were restricted to reads confidently mapped to the pathogen genome. While repeat or low-mappability regions were not explicitly masked, the use of stringent mapping quality filtering (MAPQ ≥20) helped minimize potential biases.

We collected 55 publicly available individual whole-genome sequences for *P. viticola* (NCBI: PRJNA644583 and NCBI: PRJNA329579) and 1 dataset for *P. muralis* (NCBI: PRJNA448661) from the literature.^23^^,^^31^ Among the *P. viticola* data, 54 sequenced samples were collected from Europe, and 1 was from Georgia (Figure 1A; Table S1). We considered them all to be EU samples for convenience. We mapped these data to the *P. viticola* PacBio reference genome as previously described.

For each sample, the mapping BAM file was sorted using SAMtools v1.10.^69^ The Genome Analysis Toolkit 4 (GATK v4.1.8.0) was employed to perform variant calling.^70^ The frequency of missing variants in each sample was calculated using VCFtools (v0.1.17) and samples with missing variant frequencies of more than 10% were excluded.^71^ The IBD distance in the remaining samples was calculated using PLINK (v1.90).^37^ Samples with an IBD distance higher than 0.95 were randomly selected. For the remaining 111 *P. viticola* samples, biallelic variants were filtered using VCFtools with the parameters --maf 0.05 --max-missing 0.9 --minDP 5 for further analysis. At last, 1,549,647 nuclear SNPs, 110,393 nuclear indels, and 49 mitochondrial SNPs were retained for subsequent analyses.

#### Population structure, genetic diversity, and gene flow

Mash (v2.3) was used to calculate the pairwise distances between bam-converted fastq files in all 111 *P. viticola* samples.^72^ A neighbor-joining (NJ) tree was constructed using the ape package in R v4.2.2 with the genetic distance generated using the Mash program.^73^ We performed MDS analysis based on the Mash genetic distance. SNPs from 111 *P. viticola* samples were pruned using PLINK with parameters --indep-pairwise 50 5 0.2. The pruned SNP dataset was used to identify genetic clusters using ADMIXTURE software (v.1.3).^74^

For each genomic position, the genetic diversity in a population was calculated as follows:

$$\pi =1-\left(X_{A}^{2}+X_{C}^{2}+X_{G}^{2}+X_{T}^{2}\right)$$

Where *X* is the allele frequency calculated based on the read coverage depth and was averaged across all loci in a population.^78^

Hudson’s FST was calculated as follows:

$$F_{\text{ST}}=\left(D_{\text{xy}}-\pi_{\text{xy}}\right)/D_{\text{xy}}$$

Where D_xy_ and π_xy_ represent mean sequence dissimilarity between and within populations, respectively.^79^

Tajima’s D was calculated across non-overlapping genomic windows of 5,000 bp using VCFtools (version 0.1.17).^71^ The ABBA-BABA D-statistic was computed using the Dsuite software (version 0.5 r52).^75^ Population configurations for the D-statistic analyses are detailed in Figure 3E and Table S7. Alleles were polarized using *P. muralis* as an outgroup species.

#### Analysis of the β-tubulin gene

We used aTRAM software to assemble the β-tubulin gene using sequences from our paired-end genomic library for each WGS sample.^32^ We identified four haplotypes in our Chinese and EU populations. We then combined these four haplotypes with β-tubulin genes reported in North America, EU, and Chinese populations by Fontaine et al., and conducted multiple sequence alignment using MAFFT (version 7.453) with default parameters.^76^ After removing poorly aligned regions, the maximum likelihood tree of β-tubulin sequences was constructed using IQ-TREE2 with the parameter -b 100.^77^

#### Population demographic history inference

Demographic relationships among Chinese and European populations were evaluated using fastsimcoal2 (v2.7.0) based on unfolded joint site-frequency spectra (SFS).^33^ We compared alternative three-population topologies among BHL (BJ, HN, and LN), EU4, and EU-other, and further evaluated pulse-gene-flow extensions of the best-supported no-gene-flow model. Alternative demographic scenarios for YN were also tested using BHL, YN, and EU4, with all models incorporating a YN bottleneck (Figure S4; Table S3). Multiple independent optimization runs were performed, and model fit was compared using the Akaike information criterion (AIC), with lower AIC values indicating better support. Because the SFS was conditioned on variable sites, absolute demographic times and population sizes were not interpreted.

Historical changes in effective population size were subsequently reconstructed using Stairway Plot 2 (v2.1.2).^34^ A generation time of one year per generation and a mutation rate of 3.16 × 10^−9^ mutations per site per generation, based on estimates from the related oomycete *Phytophthora ramorum*,^80^ were assumed for temporal scaling.

#### Genetic load analysis

Variant sites were annotated using ANNOVAR (2018Apr16) and SIFT4G (v.6.0).^38^^,^^39^ The gtf file for the *P. viticola* PacBio reference genome was used to build custom ANNOVAR and SIFT4G databases.^31^ Only coding-region variants were retained and classified as synonymous (Syn), nonsynonymous tolerant (Tol; SIFT >0.05), nonsynonymous deleterious (Del; SIFT <0.05), or loss-of-function (LOF; frameshifts, stop-gain/loss, splice-site changes). LOF variants were considered highly disruptive, potentially causing truncation or nonsense-mediated decay.^81^

The ancestral state of each variant was inferred using *P*. *muralis* as the outgroup, with its allele sites designated as ancestral. Derived allele frequencies were then calculated for each *P*. *viticola* population across mutation categories, based on read depth metrics from individual samples.

#### Characterization of genetic load variation across the genome

To assess genome-wide deleterious load variation among Chinese *P. viticola* populations, we divided the genome into non-overlapping 50-kb windows and quantified derived allele count of deleterious Del and LOF variants, normalizing them to EU levels. Because each window averages ∼10 genes, we randomly sampled ten genes per iteration, normalized Del and LOF loads to EU, and repeated this 1,000 times to generate an empirical null distribution. Windows with load ratios below the 5th or above the 95th percentile of this distribution were classified as having significantly reduced or increased deleterious load, respectively.

#### Genomic differentiation detection

To identify genomic regions between the LN and YN, we first partitioned the genome into non-overlapping 5 kb windows and calculated F_ST_ values. Regions with top 5% F_ST_ values and those showing the lowest 5% Tajima’s D values in either LN or YN subpopulations individually were designated as focal regions for further analysis. Within these regions, SNPs with the highest 5% F_ST_ values between LN and YN were subjected to association testing with 19 bioclimatic variables (see below). SNPs demonstrating statistically significant associations with environmental variables were classified as candidate SNPs for adaptive divergence.

#### Identification of climate-associated SNPs

We obtained global climate data at 10′ resolution from WorldClim v2,^41^ including 19 temperature- and precipitation-related bioclimatic variables. Using the R package raster, values for all variables were extracted at the sampling coordinates of 108 *P. viticola* (60 Chinese and 48 EU isolates); three EU isolates lacking geographic coordinates were excluded. For SNP–climate association analyses, climatic values were averaged at the population level. For each highly divergent SNP between the LN and YN populations, we assessed the correlation between derived allele frequencies and mean bioclimatic variables using Spearman’s rank tests. SNPs with *p* < 0.1 were retained as candidate climate-associated loci for further analysis.

#### Gene ontology enrichment analysis

GO annotations for *P. viticola* genes were retrieved from the Dataverse repository (https://doi.org/10.15454/4NYHD6). GO term enrichment analysis was performed using a hypergeometric distribution test, and Benjamini-Hochberg corrected *p*-values ≤0.05 were considered statistically significant for identifying enriched terms.

#### Standing variation analysis

SNPs shared between LN or YN and at least one other population were considered putative pre-existing variants, whereas SNPs detected only in LN or YN were classified as population-specific variants. For candidate adaptive SNPs, polymorphic site frequencies and major allele frequencies were compared with those of non-candidate SNPs using chi-square tests for site distributions and Wilcoxon rank-sum tests for allele-frequency differences.

### Quantification and statistical analysis

All statistical analyses were performed using R (version 4.2.2). Unless otherwise specified, boxplots show the median as the center line, the 25th and 75th percentiles as the lower and upper hinges, and whiskers extending to the most extreme values within 1.5 × the interquartile range (IQR). Points beyond the whiskers are plotted individually as outliers. The exact sample size (n) and the statistical unit represented by each datapoint are specified in the corresponding figure legends.

For analyses based on individual *P. viticola* isolates, the final dataset consisted of 51 EU, 26 BJ, 9 LN, 9 HN, and 16 YN isolates. Differences between European and Chinese populations in Figure 4A; Figure S8 were assessed using Wilcoxon rank-sum tests. Comparisons among Chinese subpopulations in Figure 4A were evaluated using Kruskal–Wallis tests followed by post hoc pairwise comparisons.

For analyses based on genomic windows, candidate SNPs, or genes, each datapoint represents one genomic window, SNP, or gene, respectively, as indicated in the corresponding figure legend. Pairwise comparisons in Figure 5; Figures S9, and S14–S16 were performed using Wilcoxon rank-sum tests. Statistical significance is indicated as ∗∗∗: *p* < 0.001, ∗∗: *p* < 0.01, and ∗: *p* < 0.05.

## Supplemental information

Document S1. Figures S1–S16

Table S1. Sample information for 123 *Plasmopara viticola* isolates and one *P. muralis* isolate analyzed in this study, related to Figures 1 and S1

Table S2. Details of 49 mitochondrial SNPs identified among *P. viticola* populations, related to Figure S2

Table S3. Summary of demographic model comparisons, related to Figure 2C and Figure S4

Table S4. Population genetic diversity of *P. viticola* populations, related to Figure 3A

Table S5. Pairwise FST values among *P. viticola* populations, related to Figure 3B

Table S6. Pairwise FST values used for isolation-by-distance analyses across 15 sampling sites in China (*n* = 9 sites) and Europe (*n* = 6 sites), related to Figure 3D

Table S7. Summary of ABBA-BABA D-statistics among *P. viticola* populations using Dsuite, related to Figure 3E
