# Supplementary material for: Standing genetic variation facilitates climatic adaptation in the invasive grapevine pathogen Plasmopara viticola under demographic bottlenecks
Source: iScience. 2026 Oct 14;29(11):117847. doi: 10.1016/j.isci.2026.117847 (PMC13694879; doi:10.1016/j.isci.2026.117847)
Supplement: Supplementary file 1 [file mmc1.pdf]

iScience, Volume *29*

Supplemental information

Standing genetic variation facilitates climatic adaptation in the invasive grapevine pathogen *Plasmopara viticola* under demographic bottlenecks

Xuncheng Wang, Yonghua Li, Junbo Peng, Wei Zhang, Ishara S. Manawasinghe, Qikai Xing, Hui Wang, Mei Liu, Ying Zhou, Linna Wu, Jianping Xu, Xinghong Li, and Jiye Yan

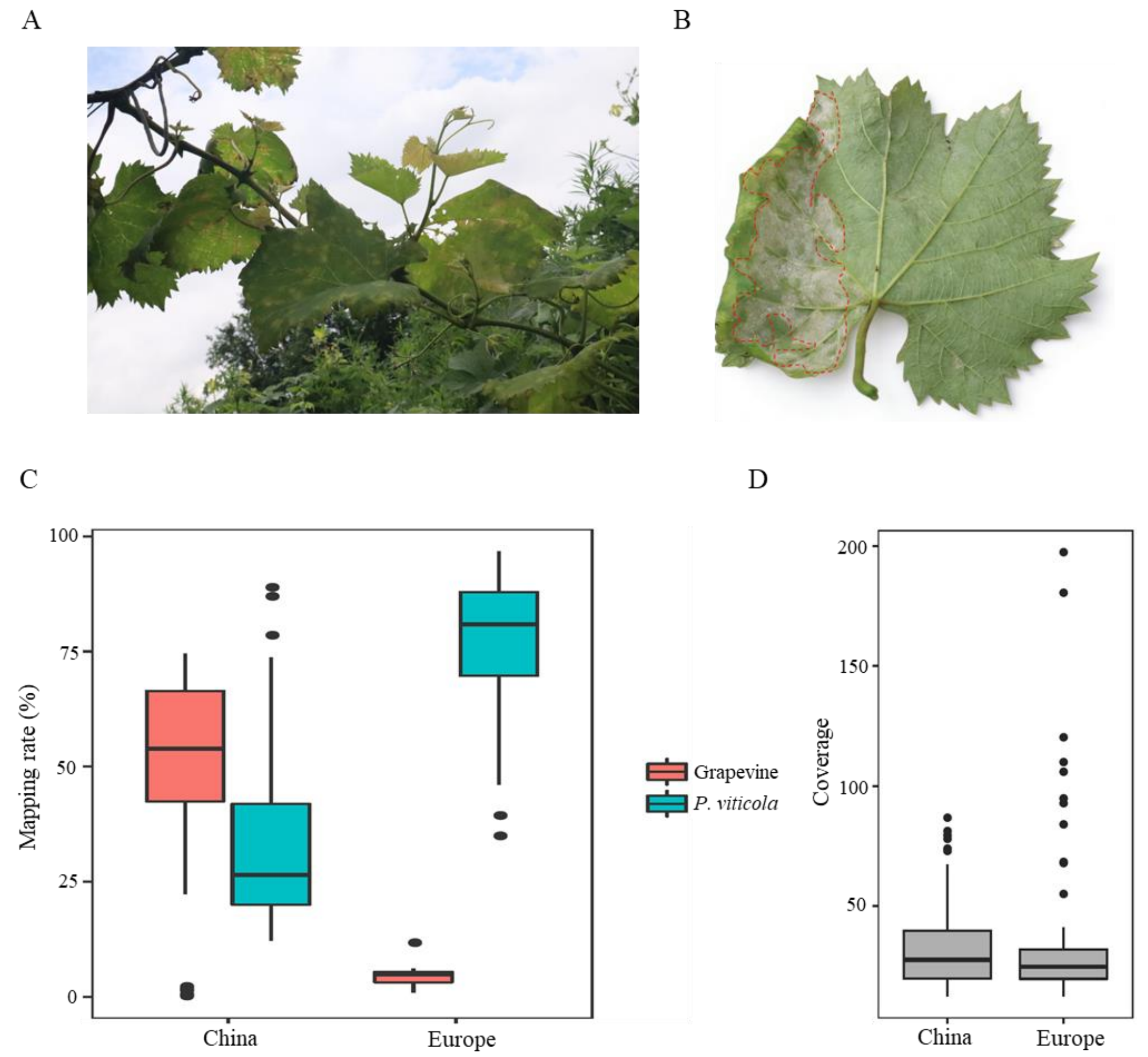

Figure S1. Field symptoms of grapevine downy mildew and sequencing/mapping characteristics of lesion samples

(A) Downy mildew-infected grapevine leaves on shoots in the field. (B) Symptomatic leaf used for sampling; the region outlined by the red dashed box indicates lesion tissue excised for sequencing. (C) Proportions of sequencing reads mapped to the host and pathogen genomes. Each datapoint represents one isolate (China, n = 60; Europe, n = 51). (D) Genome coverage of reads mapped to the *P. viticola* reference genome. Each datapoint represents one isolate (China, n = 60; Europe, n = 51). For all boxplots, the center line indicates the median, boxes indicate the 25th–75th percentiles, whiskers extend to 1.5 × IQR, and points beyond the whiskers are shown individually.

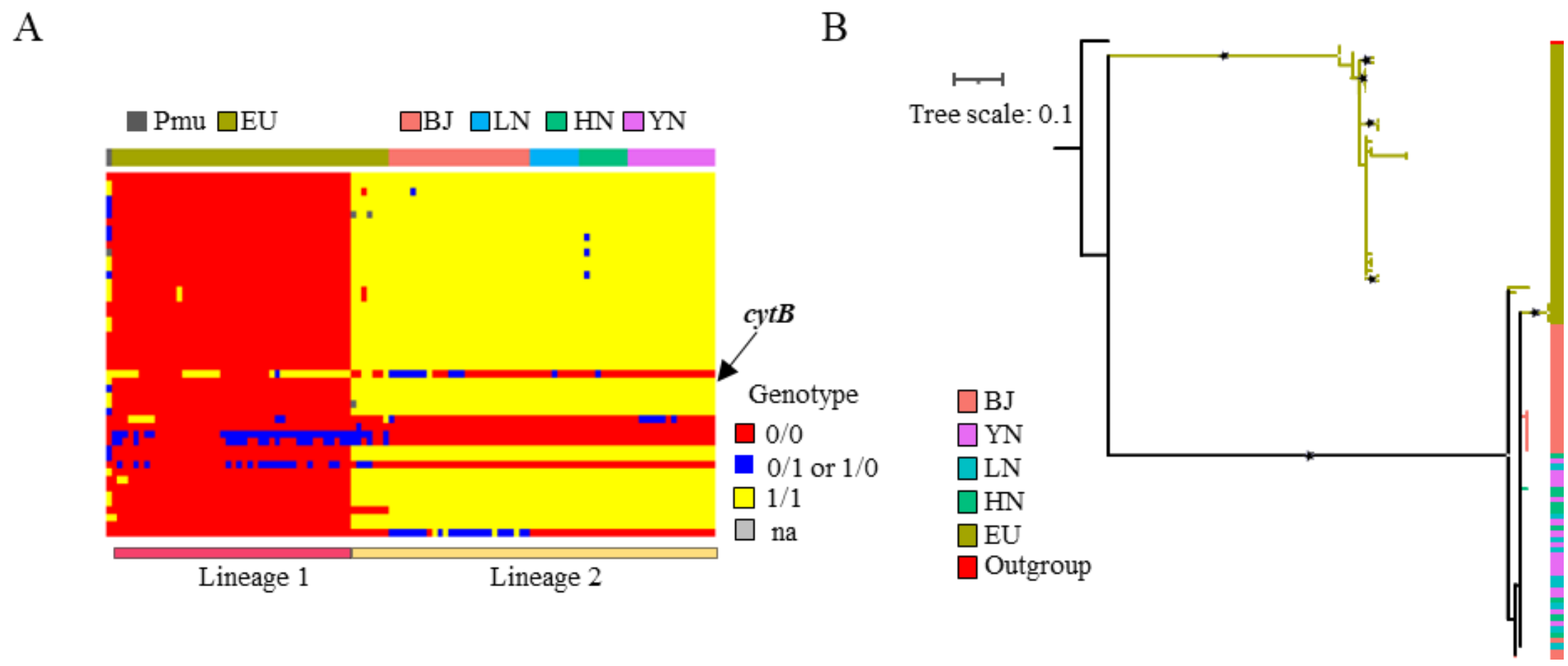

Figure S2. Analysis of mitochondrial variants in *P. viticola*

(A) A total of 49 mitochondrial single nucleotide polymorphisms (SNPs) were identified in *P. viticola* WGS samples. In the heatmap, the horizontal lines represent SNPs, and the vertical lines represent individual samples. The arrow indicates SNPs in the cytochrome B (*cytB*) gene, corresponding to fungicide-resistant samples with amino acid A at position 143 in cytB. Red cells (genotype 0/0) indicate the reference allele; Yellow cells (genotype 1/1) indicate the alternative allele, corresponding to fungicide-sensitive samples with amino acid G at position 143 in cytB; Blue cells (genotypes 0/1 or 1/0) represent heterozygous sites; na: missing data. (B) Phylogenetic analysis of *P. viticola* isolates from Europe and China based on WGS data. A ML tree was constructed using 49 SNPs derived from mitochondrial genome in the WGS dataset, and rooted using SNPs from *P. muralis* as an outgroup. Asterisks denote internal nodes with bootstrap support values exceeding 85%.

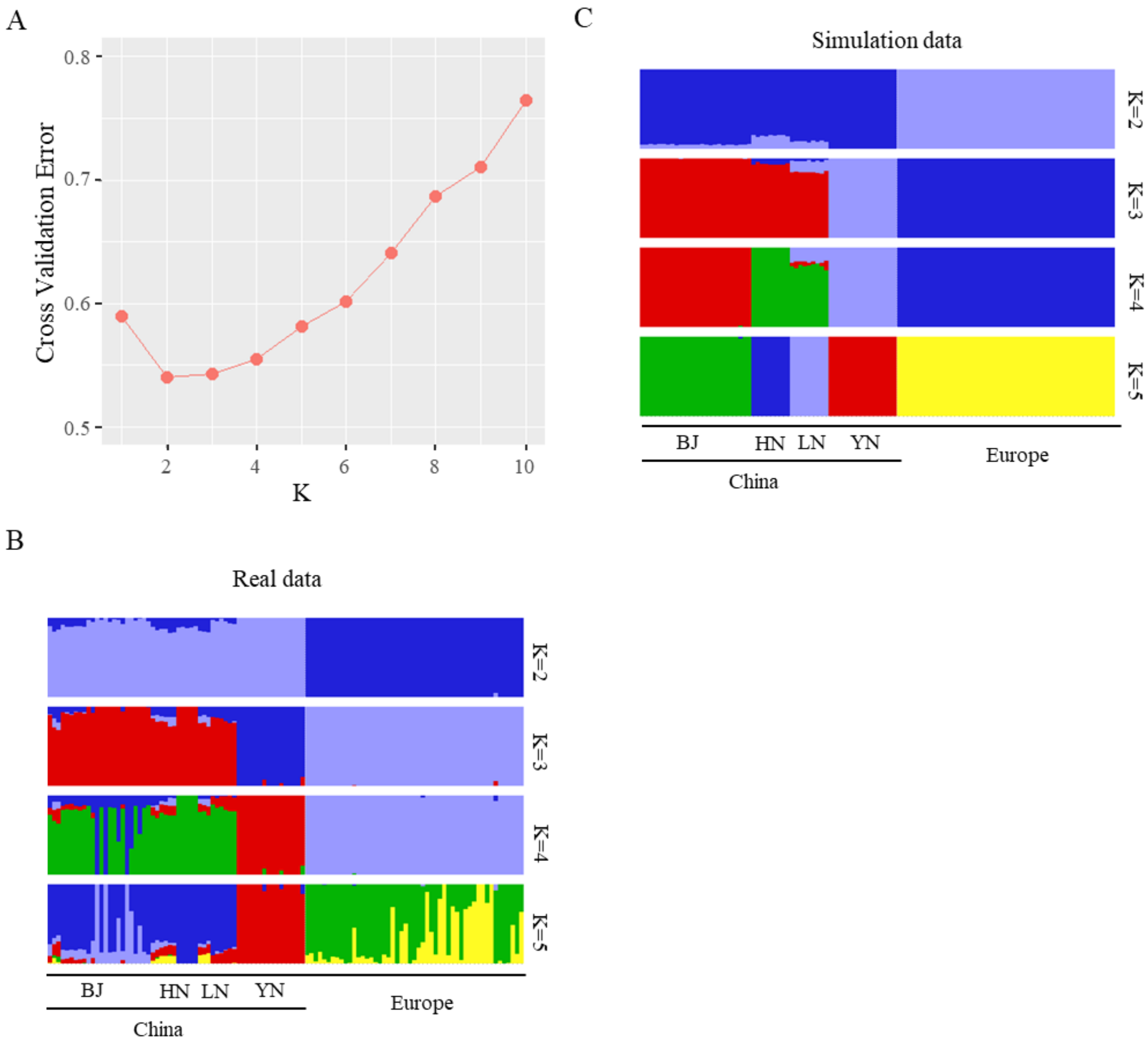

Figure S3. Admixture analysis of *P. viticola*

(A) Cross-validation error across different K values. (B) Real data: real genotype data from the samples. (C) Simulation data: simulated genotype data for each *P. viticola* population. To determine the influence of analyzing genotypes from pooled DNA, equal amounts of individual genotypes were separately simulated based on the number of samples in BJ, LN, HN, YN, and EU populations and subjected to ADMIXTURE analysis. Briefly, for each SNP, the average allele frequency for each population was calculated using the read counts from the AD tags in the vcf file and used to simulate a number of genotypes equal to the number of samples in the population under Hardy–Weinberg equilibrium. Simulated SNPs in pruned positions identified in real data were then analyzed using ADMIXTURE.

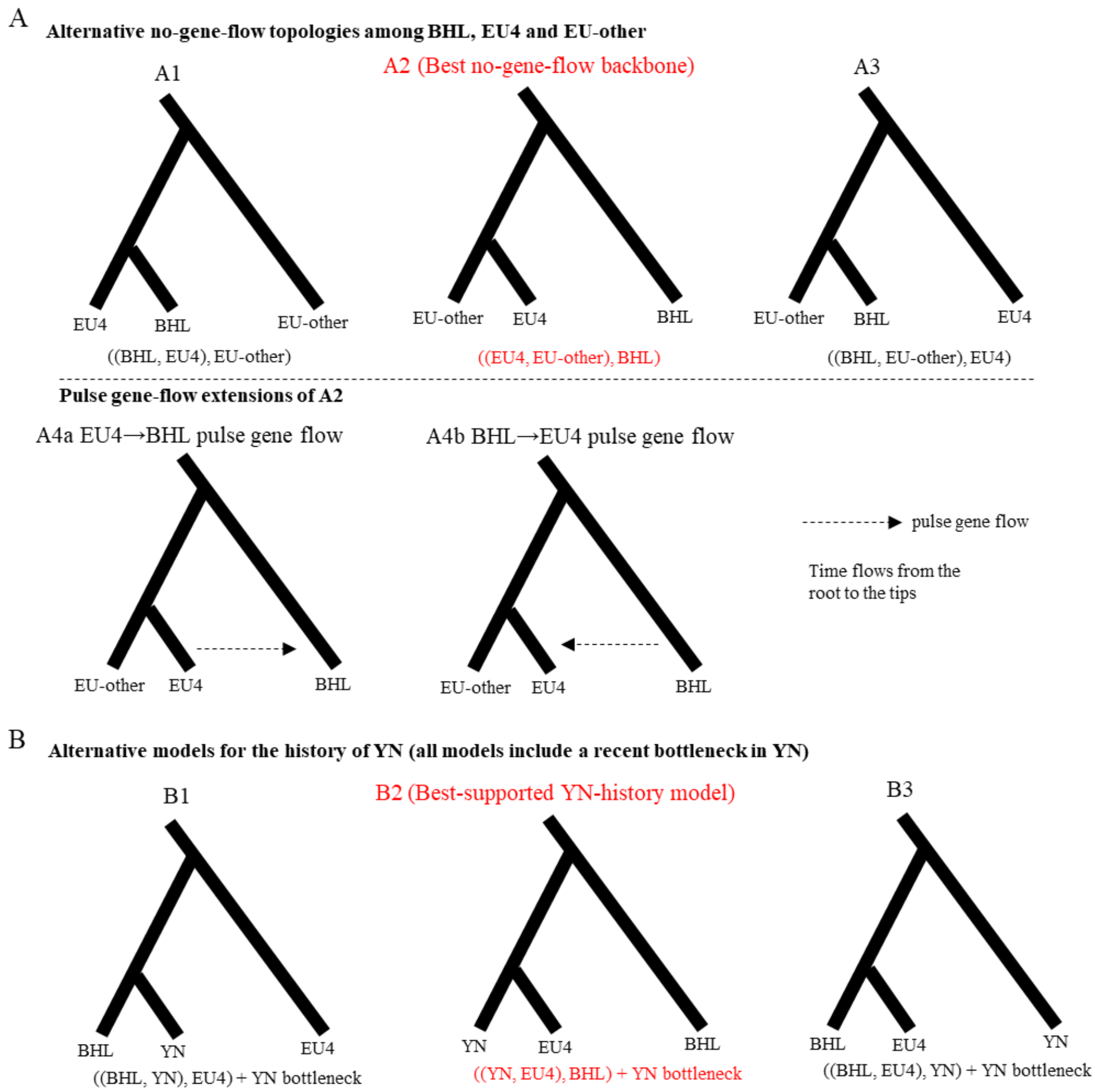

Figure S4. Demographic models evaluated using fastsimcoal2

(A) Alternative no-gene-flow topologies among BHL, EU4, and EU-other (A1–A3), together with two pulse gene-flow extensions of the best-supported backbone model A2 (A4a and A4b). Dashed arrows indicate the forward-time direction of the modeled pulse gene-flow event. (B) Alternative models for the demographic history of YN (B1–B3). All three models include a recent bottleneck in YN but differ in the inferred relationship among YN, BHL, and EU4. A2 and B2 were the best-supported models within their respective no-gene-flow model sets. Model-selection statistics are provided in Table S3.

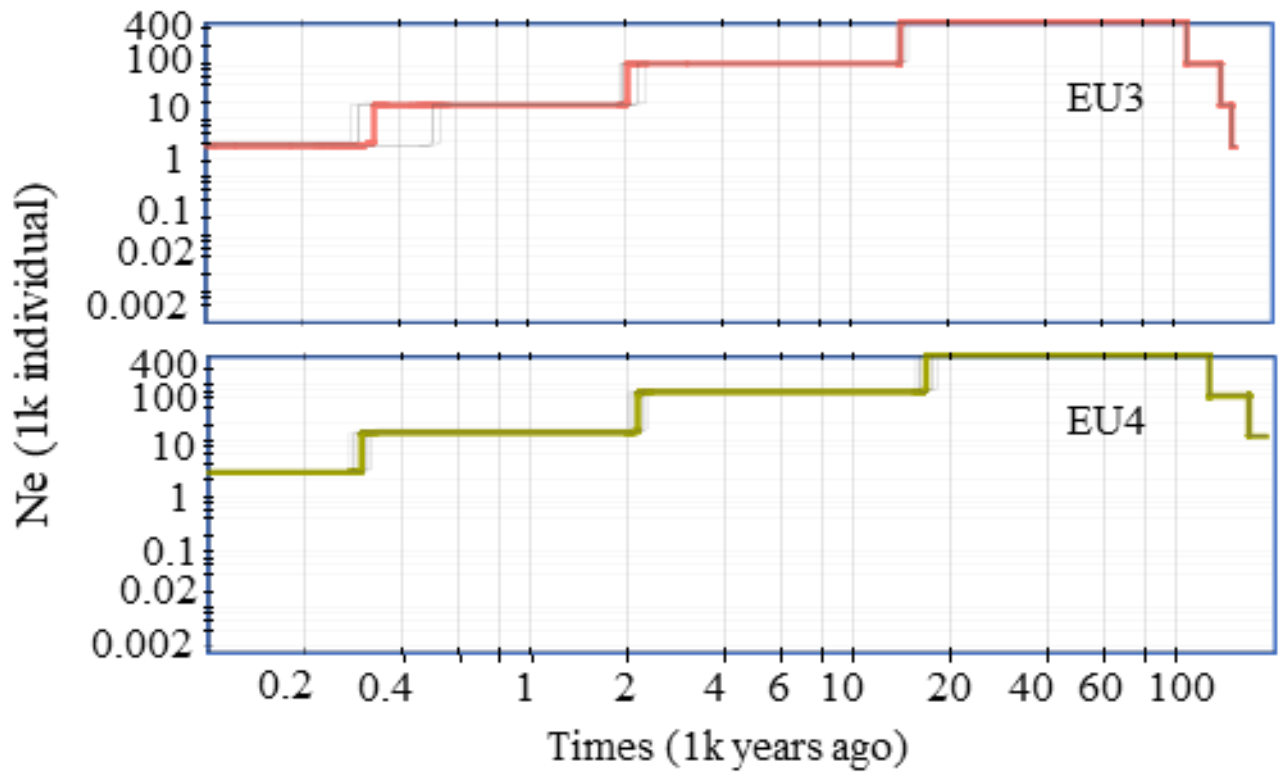

Figure S5. Population demographic history of *P. viticola* in European subpopulations (EU3 and EU4). Here, we only focused on the EU3 and EU4 subpopulations within EU, as their clustering was strongly supported by high bootstrap values in the nuclear phylogenetic tree.

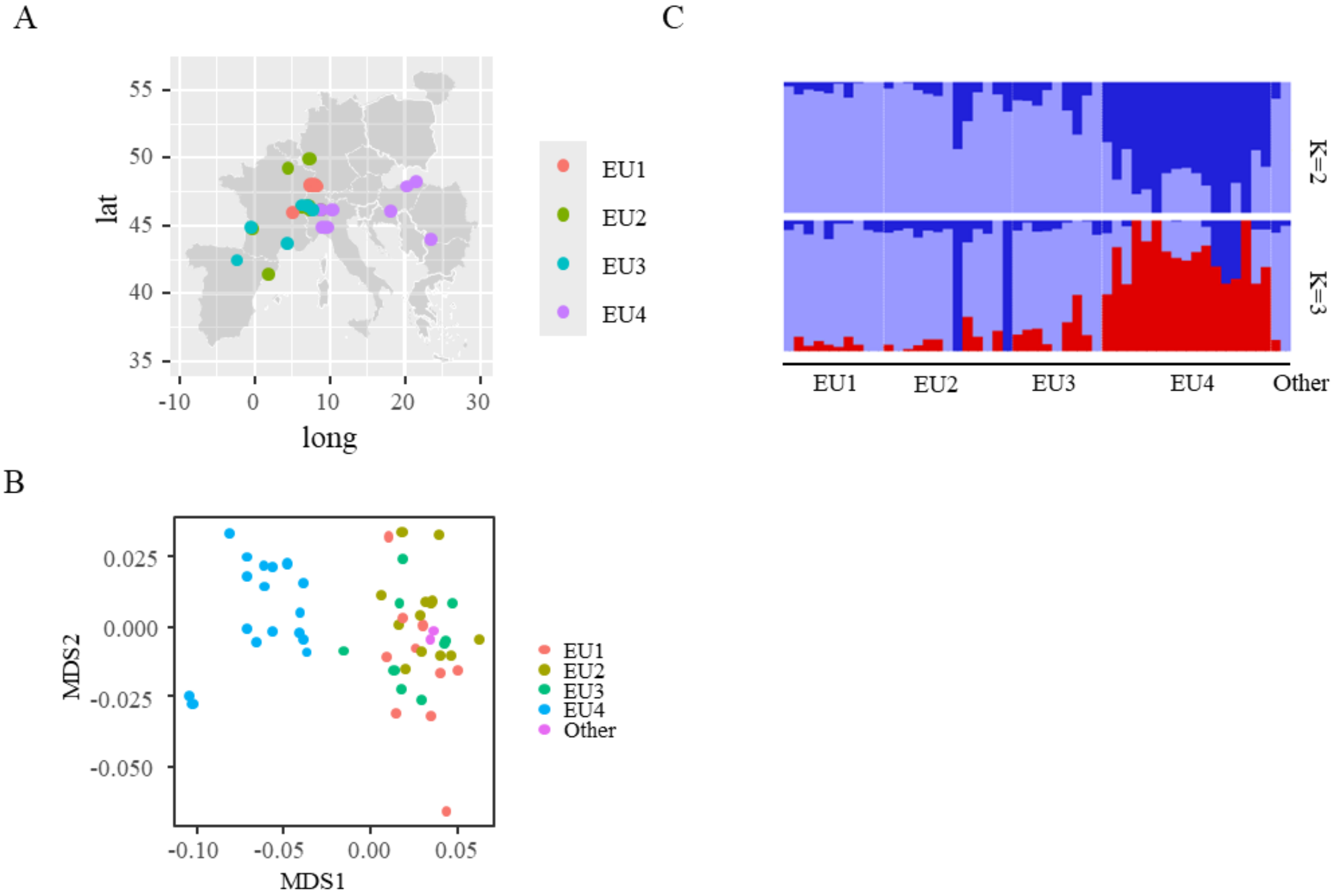

Figure S6. Population structure analysis of *P. viticola* in EU

(A) Geographic distribution of EU subpopulations. EU1-EU4 denote EU subpopulations delineated based on phylogenetic clustering observed in Figure 1C. (B) Multidimensional scaling (MDS) analysis of *P. viticola* EU samples based on the MASH genetic distance. (C) ADMIXTURE analysis of *P. viticola* EU samples based on genotype data.

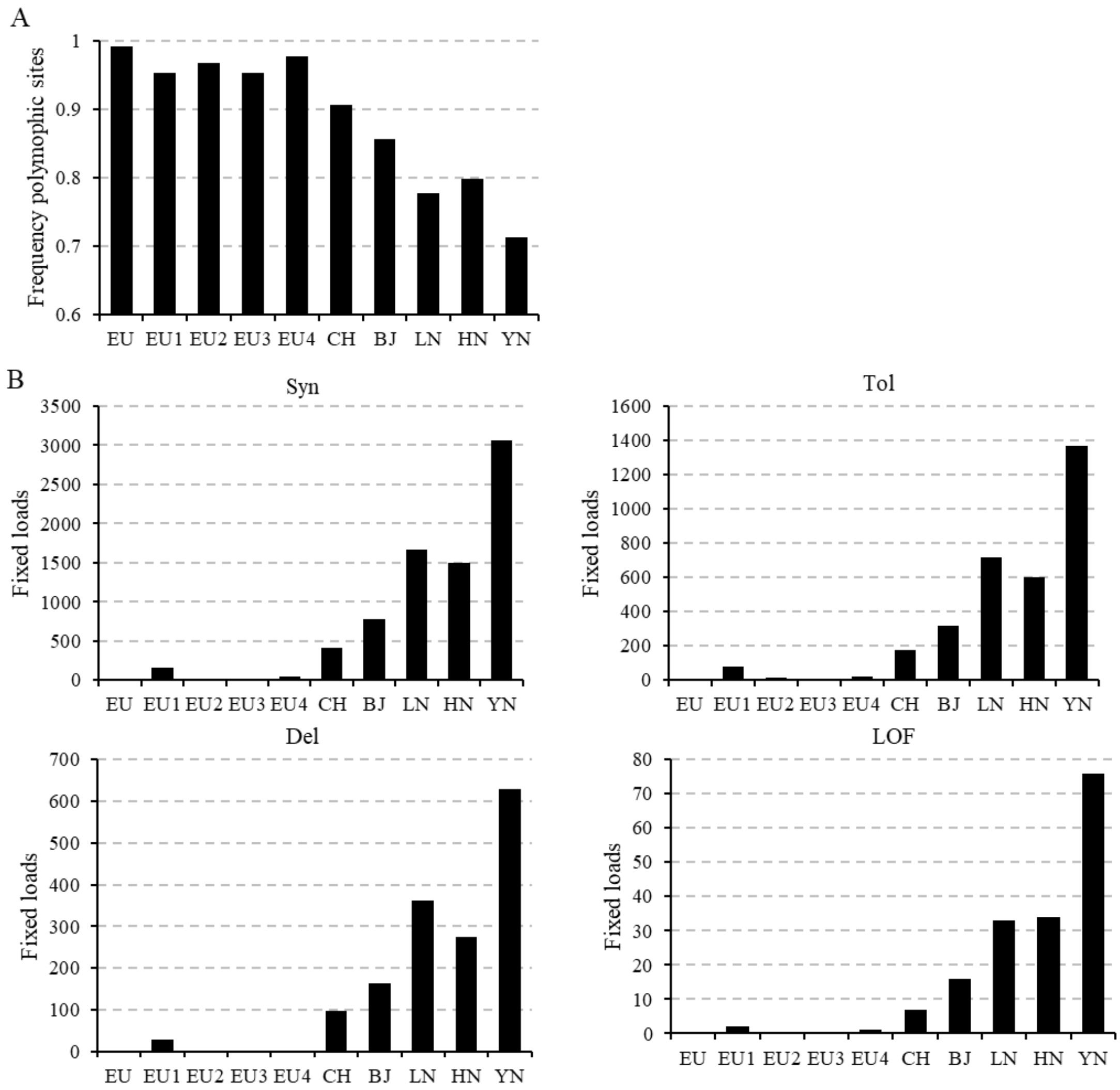

Figure S7. Characterization of polymorphic sites and homozygous fixed genetic load in *P. viticola*

(A) The frequency of polymorphic sites across different populations of *P. viticola*. (B) The homozygous fixed genetic load in *P. viticola* across four mutation categories.

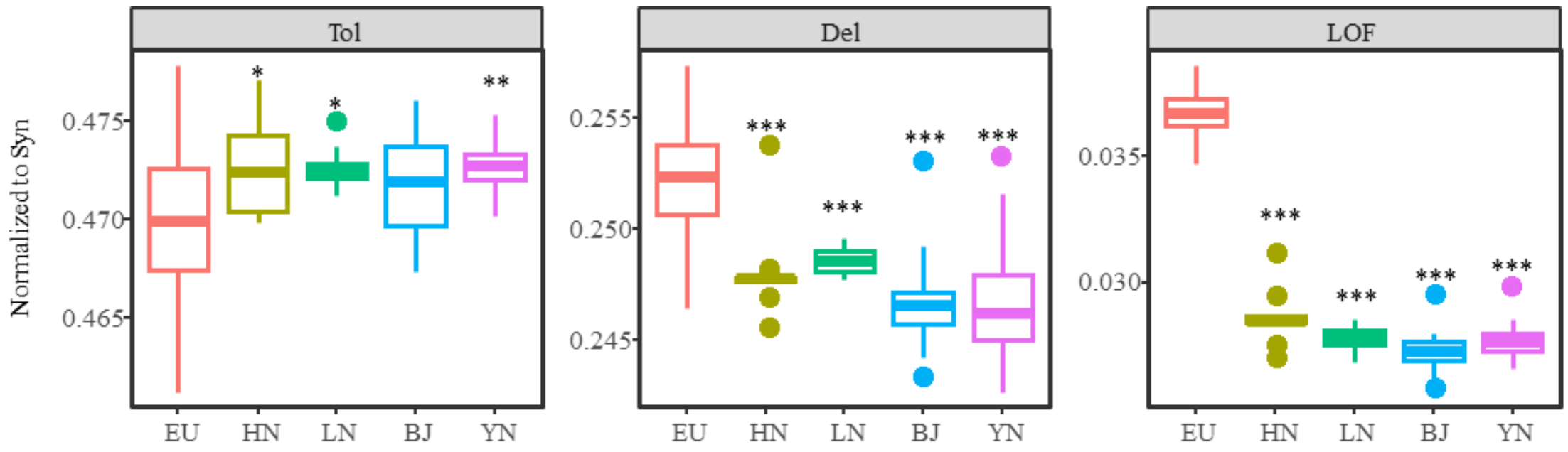

Figure S8. Normalized derived allele loads in *P. viticola*. Derived mutation loads were normalized to synonymous (Syn) mutation loads. Each datapoint represents one isolate (EU, n = 51; HN, n = 9; LN, n = 9; BJ, n = 26; YN, n = 16). Boxplots show the median as the center line, the 25th and 75th percentiles as the lower and upper hinges, and whiskers extending to values within 1.5 × the interquartile range (IQR). Differences between EU and each Chinese subpopulation were assessed using Wilcoxon rank-sum tests. ***: $P < 0.001$; **: $P < 0.01$; *: $P < 0.05$.

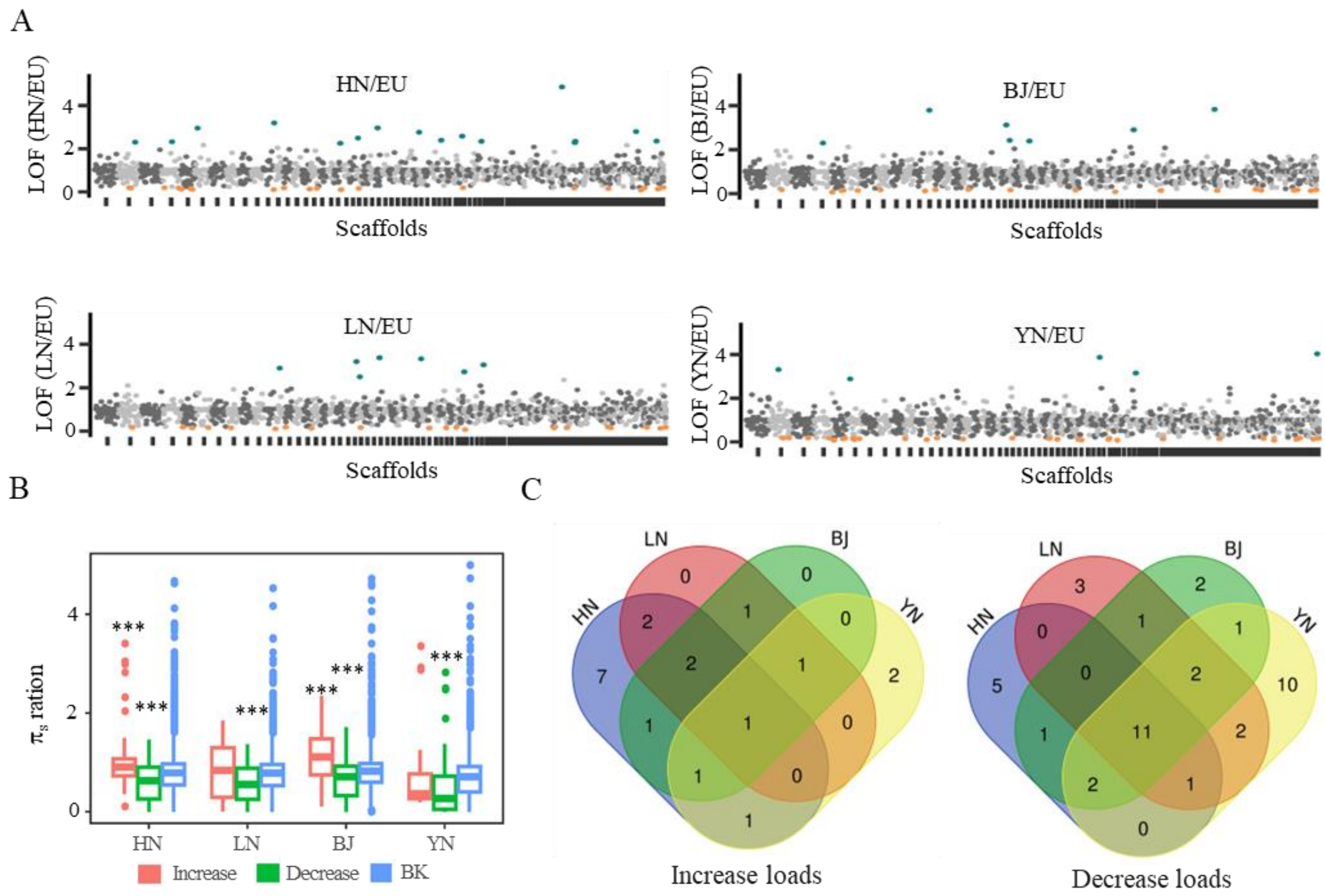

Figure S9. Genome-wide variation in deleterious mutation loads across Chinese populations

(A) Genome-wide variation of LOF mutation loads across Chinese populations. For each 50 kb non-overlapping genomic window across the genomes in Chinese populations, derived allele counts of deleterious mutations were normalized against those in EU samples. (B) Synonymous nucleotide diversity ratios (π_s ratios) of 50-kb genomic windows with increased or reduced LOF loads and background windows (BK). Each datapoint represents one 50-kb genomic window. Boxplot elements are defined as above. The numbers of reduced-load windows were BJ, n = 20; LN, n = 20; HN, n = 20; YN, n = 29, and the numbers of increased-load windows were BJ, n = 7; LN, n = 7; HN, n = 15; YN, n = 6. Boxplots show the median as the center line, the 25th and 75th percentiles as the lower and upper hinges, and whiskers extending to values within 1.5 × the interquartile range (IQR). Increased- and reduced-load windows were separately compared with background windows using Wilcoxon rank-sum tests. ***$P < 0.001$; **$P < 0.01$; *$P < 0.05$. (C) Venn diagram analysis of genomic regions in Chinese populations that showing significantly elevated or reduced LOF mutation loads in Chinese *P. viticola* vs. EU samples.

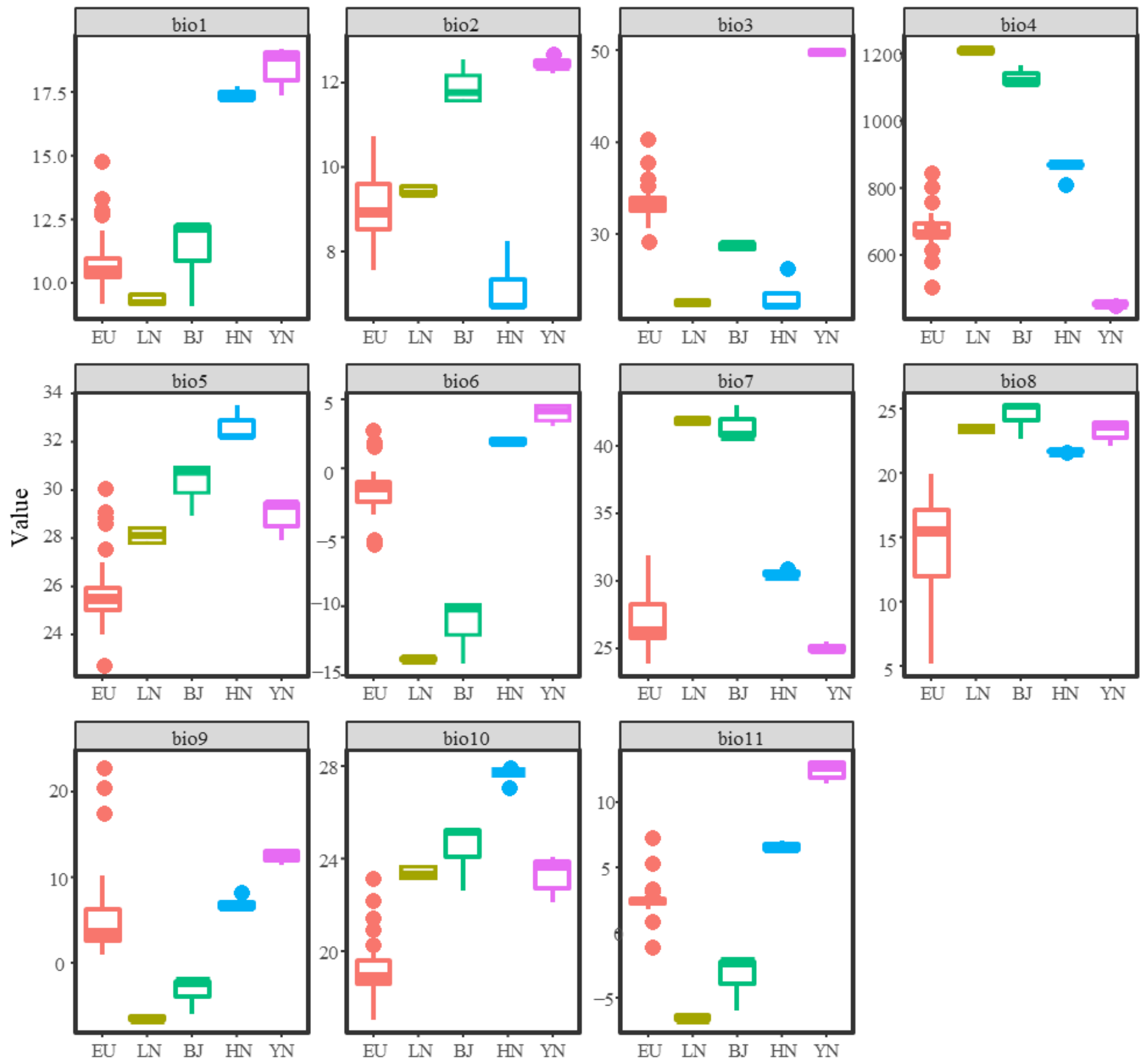

Figure S10. Temperature-related bioclimate variables. Each datapoint represents the WorldClim value extracted at the sampling coordinate of one isolate (EU, n = 48; LN, n = 9; BJ, n = 26; HN, n = 9; YN, n = 16); three EU isolates lacking geographic coordinates were excluded. Boxplots show the median, 25th–75th percentiles, and 1.5 × IQR whiskers. bio1 = Annual Mean Temperature; bio2 = Mean Diurnal Range (Mean of monthly (max temp - min temp)); bio3 = Isothermality (bio2/ bio7) (×100); bio4 = Temperature Seasonality (standard deviation ×100); bio5 = Max Temperature of Warmest Month; bio6 = Min Temperature of Coldest Month; bio7 = Temperature Annual Range (bio5-BIO6); bio8 = Mean Temperature of Wettest Quarter; bio9 = Mean Temperature of Driest Quarter; bio10 = Mean Temperature of Warmest Quarter; bio11 = Mean Temperature of Coldest Quarter

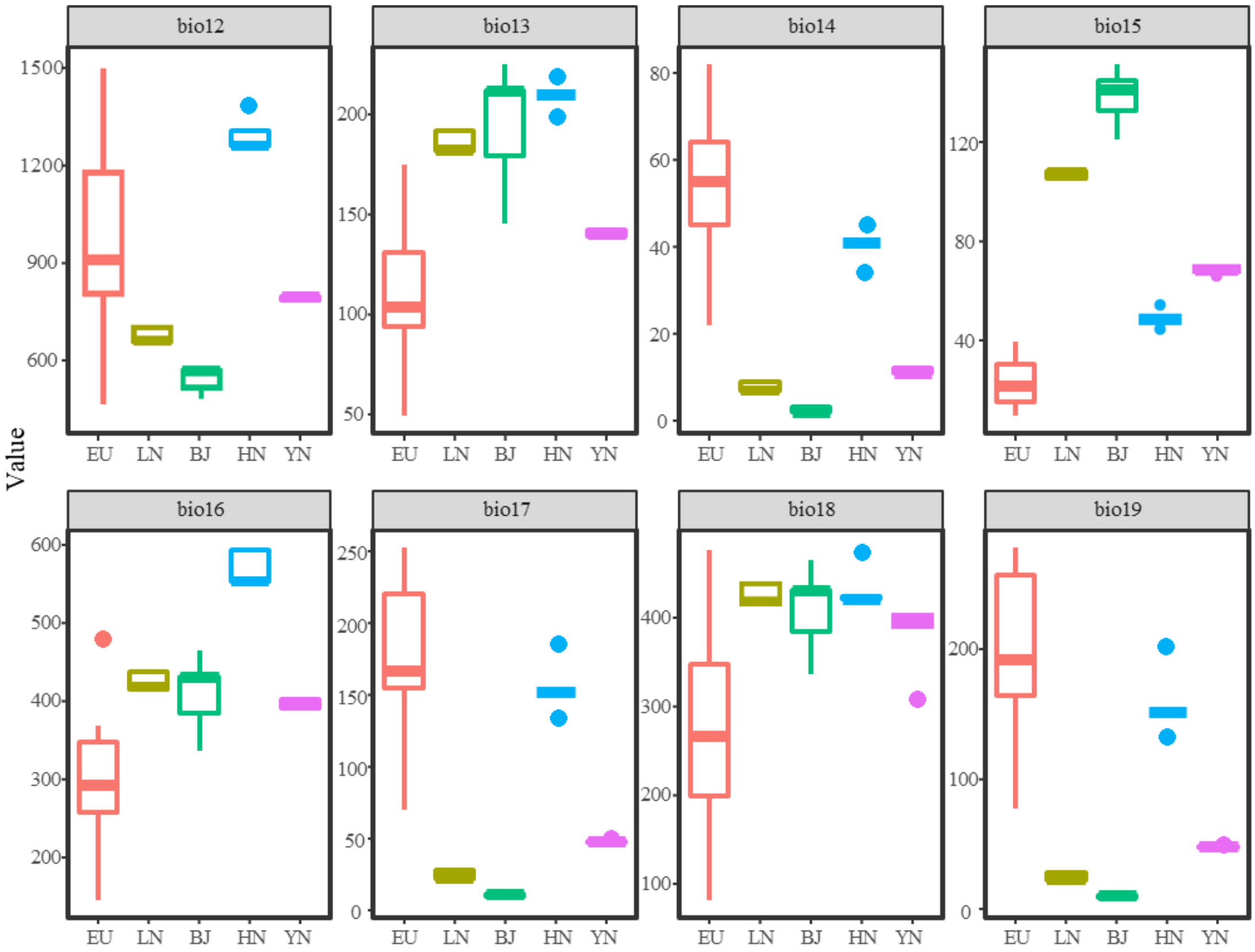

Figure S11. Precipitation-related bioclimate variables. Each datapoint represents the WorldClim value extracted at the sampling coordinate of one isolate (EU, n = 48; LN, n = 9; BJ, n = 26; HN, n = 9; YN, n = 16); three EU isolates lacking geographic coordinates were excluded. Boxplots show the median, 25th–75th percentiles, and 1.5 × IQR whiskers. bio12 = Annual Precipitation; bio13 = Precipitation of Wettest Month; bio14 = Precipitation of Driest Month; bio15 = Precipitation Seasonality (Coefficient of Variation); bio16 = Precipitation of Wettest Quarter; bio17 = Precipitation of Driest Quarter; bio18 = Precipitation of Warmest Quarter; bio19 = Precipitation of Coldest Quarter

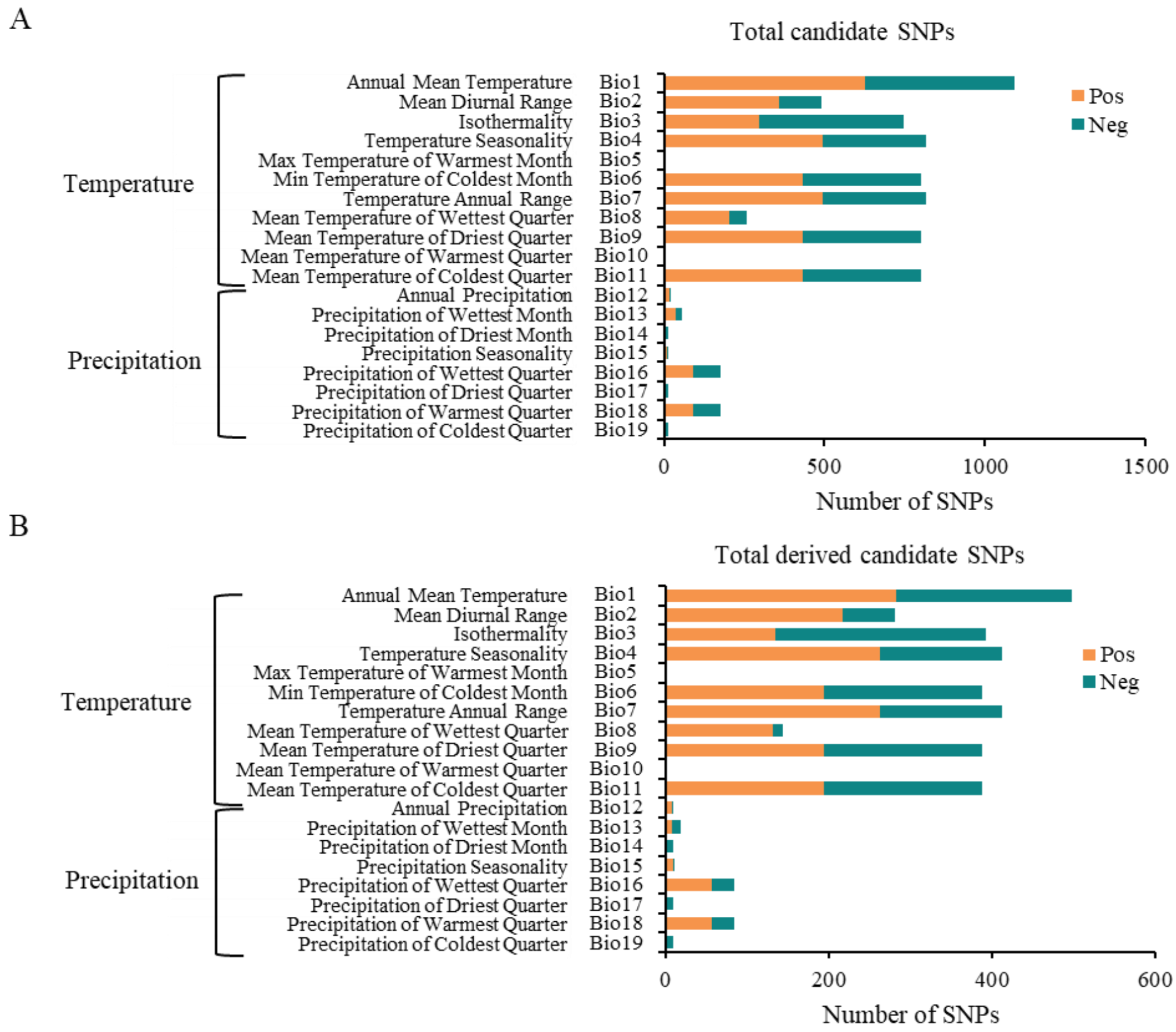

Figure S12. Association of candidate adaptive SNPs with 19 bioclimate variables

(A) Total number of candidate SNPs significantly associated with 19 bioclimatic variables. (B) Number of candidate SNPs with ancestrally inferred alleles (from *P. muralis*) significantly associated with 19 variables. Pos: variants with derived alleles positively correlated with the climatic variable (Spearman correlation, $P < 0.1$); Neg: variants with derived alleles negatively correlated with the climatic variable.

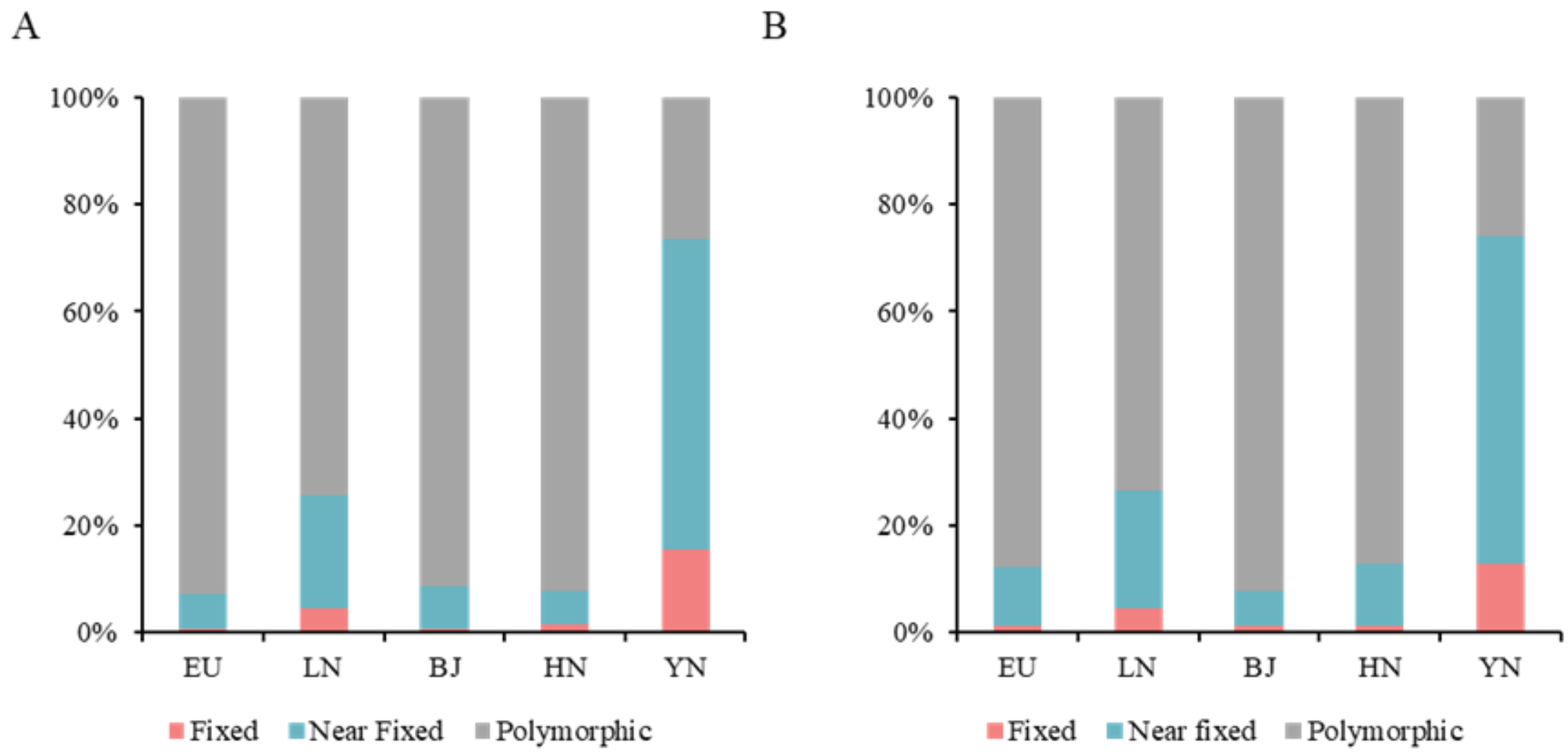

Figure S13. Proportions of candidate adapted SNPs with fixed alleles or nearly fixed alleles

(A) Total candidate adaptive SNPs. (B) Del and LOF candidate adaptive SNPs.

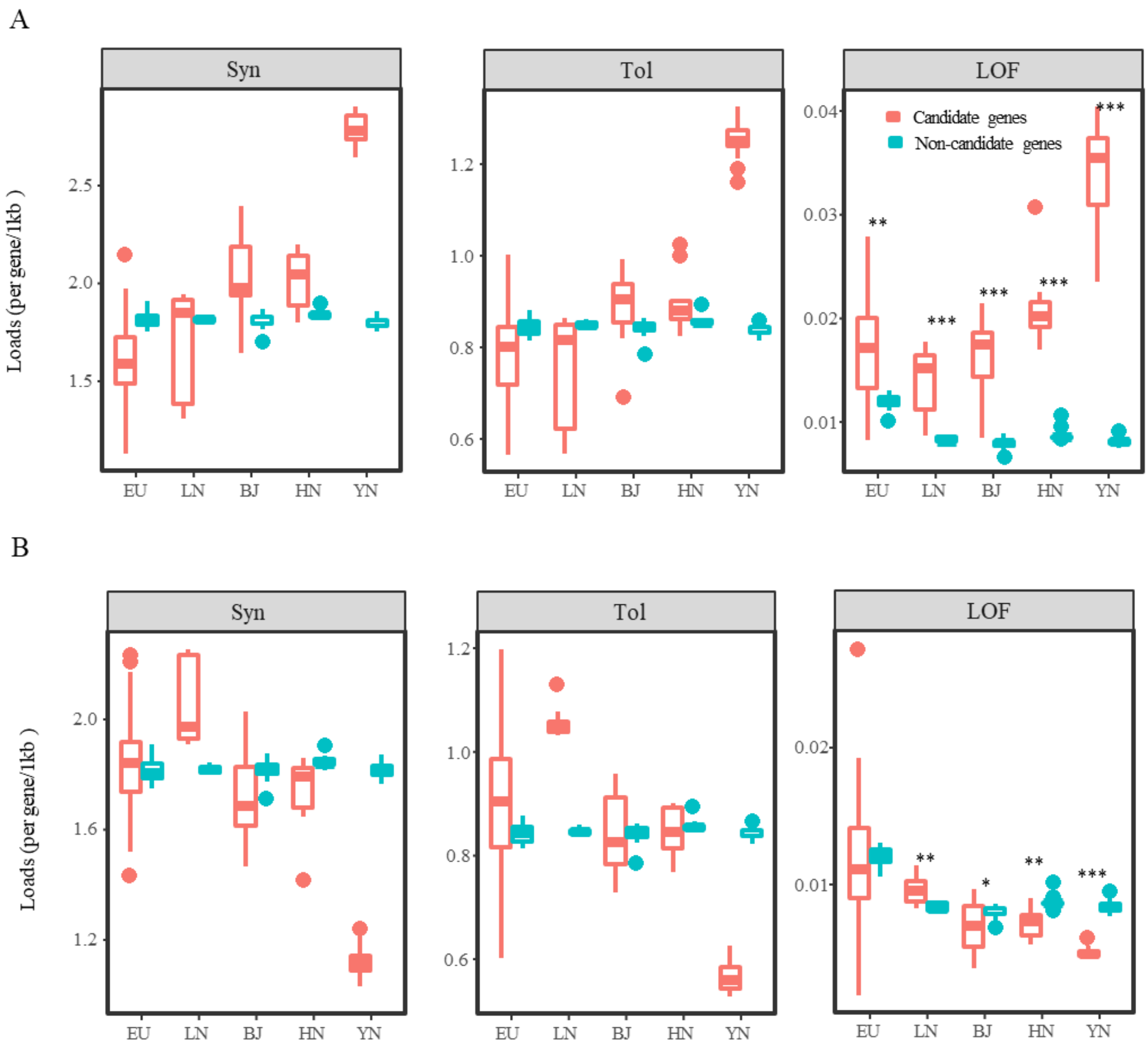

Figure S14. Boxplot analysis of the derived mutation load in candidate adaptive genes

(A) Genetic load profile in positively associated candidate adaptive genes. A gene was classified as a positively correlated candidate gene when its most divergent SNP locus between LN and YN demonstrated a positive correlation between derived allele frequencies and the bio1 climatic variable. (B) Genetic loads profile in negatively associated candidate genes. A gene was classified as a negatively correlated candidate gene when its most divergent SNP locus between LN and YN demonstrated a negative correlation between derived allele frequencies and the bio1 climatic variable. Each datapoint represents one isolate (EU, n = 51; LN, n = 9; BJ, n = 26; HN, n = 9; YN, n = 16). Boxplots show the median, 25th–75th percentiles, and 1.5 × IQR whiskers. Candidate and non-candidate genes within each population were compared using Wilcoxon rank-sum tests. ****P* < 0.001; ***P* < 0.01; **P* < 0.05.

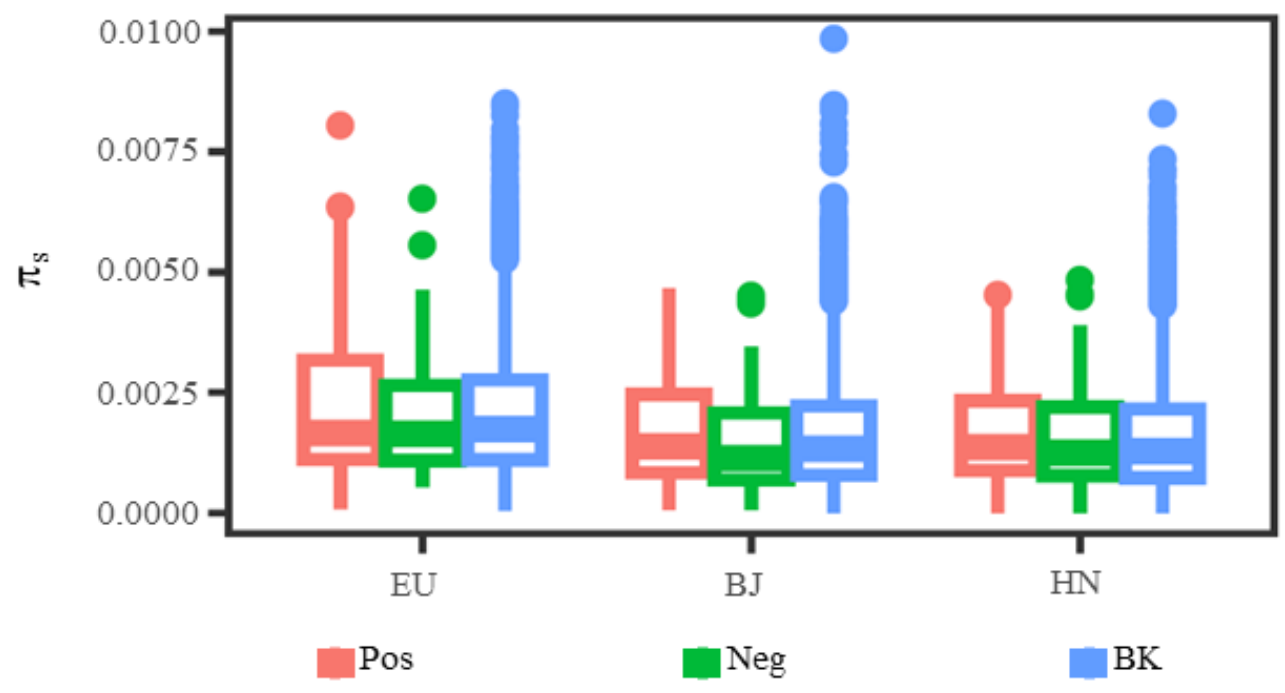

Figure S15. Local synonymous nucleotide diversity associated with candidate adaptive genes in *P. viticola*. Synonymous nucleotide diversity (πs) was calculated in non-overlapping 5-kb genomic windows. Windows containing candidate genes whose most highly differentiated SNPs between LN and YN showed positive (Pos) or negative (Neg) associations with bio1 were classified accordingly; BK denotes windows containing non-candidate background genes. Each datapoint represents one unique 5-kb genomic window (Pos, n = 62; Neg, n = 57; BK, n = 5,051). Boxplots show the median as the center line, the 25th and 75th percentiles as the lower and upper hinges, and whiskers extending to values within 1.5 × the interquartile range (IQR).

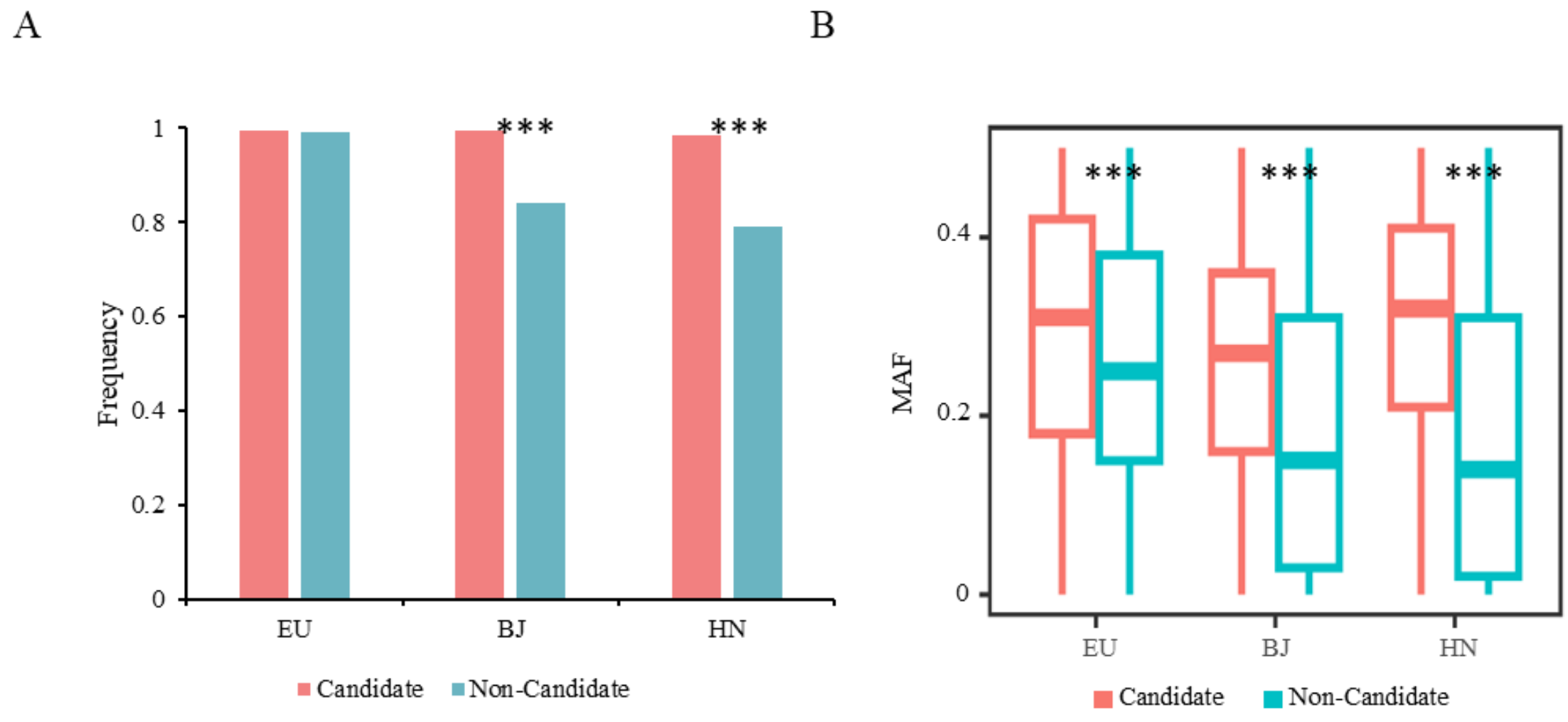

Figure S16. Analysis of standing genetic variation in candidate adaptive SNPs

(A) Comparison of polymorphic-site frequencies between deleterious candidate adaptive SNPs and non-candidate SNPs across EU, BJ, and HN populations. The candidate set comprised 155 SNPs annotated as deleterious missense (Del) or loss-of-function (LOF) variants, and 10,000 non-candidate SNPs were randomly selected as the background set. Differences in polymorphic-site frequencies between the two groups were assessed using χ^2 tests. (B) Minor allele frequency (MAF) distributions of Del or LOF candidate adaptive SNPs and randomly selected non-candidate SNPs. Each datapoint represents one SNP (candidate, n = 155; non-candidate, n = 10,000). Differences between candidate and non-candidate SNPs within each population were assessed using Wilcoxon rank-sum tests. Boxplots show the median as the center line, the 25th and 75th percentiles as the lower and upper hinges, and whiskers extending to values within 1.5 × the interquartile range (IQR). ***: $P < 0.001$.
